# On the Elemental Impact Factor, a Method to Determine an Alloy’s Compositional Influences upon Phase Stability and Metallurgical Material Properties

**DOI:** 10.3390/ma13245747

**Published:** 2020-12-16

**Authors:** Danielle L. Cote, Bryer C. Sousa, Victor K. Champagne, Richard D. Sisson

**Affiliations:** 1 Materials Science and Engineering Program, Department of Mechanical Engineering, Worcester Polytechnic Institute, 100 Institute Road, Worcester, MA 01609, USA; sisson@wpi.edu; 2 U.S. Army Research Laboratory, Aberdeen Proving Ground, Adelphi, MD 21005-5201, USA; victor.k.champagne.civ@mail.mil

**Keywords:** elemental impact factor, materials design, alloy composition, computational thermodynamics and kinetics, phase formation, metallurgical properties and microstructure, metastability

## Abstract

Design-driven materials engineering is gaining wider acceptance with the advancement and refinement of commercially available thermodynamic software as well as enhanced computing power. Computationally designed materials are a significant improvement over the more common and resource-intensive experimental approach to materials design by way of trial and error. While not entirely eliminating experimental methods for alloy design, thermodynamic and kinetic models provide accurate predictions of phases within a given alloy, which enables material properties to be calculated. Accordingly, the present paper introduces a new technique that offers a systematic method of material design by way of utilizing commercial computational software, which has been termed the elemental impact factor. In turn, the present manuscript considers Al 6061 as a proof-of-concept metallic alloy system for elemental impact factor substantiation. Effects of chemical composition on resultant equilibrium and metastable material phases as well as properties can be efficiently assessed with the elemental impact factor framework for metallurgical materials design. Desired phases or properties may be produced by adding elements with a positive elemental impact factor, while deleterious phases or undesired properties may be reduced by adding elements with a negative elemental impact factor. Therefore, the elemental impact factor methodology was presented and then demonstrated herein with examples that showcase the technique’s potential applications and utility for integrated structure-processing-property-performance analysis.

## 1. Introduction, Background and Motivation

In 2011, the Materials Genome Initiative (MGI) was formalized so that the “development of advanced materials can be accelerated through advances in computational techniques,” as stated in [1]. Thus, the MGI is directly applicable to materials design. From a historical perspective, innovative materials were designed using scientific intuition developed by experts over time and trial-and-error experimentation. Before the development of functional materials software modeling packages, or engineering friendly computer coding languages, such as Fortran, a number of researchers employed hand-written analytical methods to crudely model and predict the behavior of alloys. A more common approach, however, was the time- and cost-ineffective and inefficient use of a trial-and-error modality to design novel alloys. Computational software packages have since augmented many Edisonian operations, and materials are being developed at a significantly faster pace and at a lower cost. With the rising availability of computers and computing capacity, materials databases were digitalized, and computer codes replaced manual calculations. At present, with the increase in computational power, sophisticated computational thermodynamic and kinetic software packages are available, which have accelerated the development of novel materials and do so at lower costs, as detailed in [2,3,4,5].

While one part of the MGI was to develop a more efficient portal to share data, another area was the rapid innovation of novel materials by computational modeling. Currently, new materials are designed and developed using the following stages: discovery, development, property optimization, systems design and integration, certification, manufacturing, and deployment (including sustainment and recovery), as discussed in [1]. As indicated above, this is an inefficient and often ineffective method for modern materials development. The computational materials science and engineering approach outlined in the MGI was proposed to shorten the new material development lifetime from ten or twenty years to two or three years [1]. The MGI report specifically notes the use of modeling to develop “lightweight protection materials”. Accordingly, in this work, computational thermodynamic, kinetic, and solidification models were utilized to explore a novel method that will help with the design of alloys for the specific purpose cited above as a proof-of-concept example. Extensive work in the area of materials design and development has been performed by G. Olson at Northwestern University (Chicago, IL, USA) and QuesTek Innovations LLC (Chicago, IL, USA) [6,7,8]. An example of successful materials by design follows from the work of Olson and others, wherein Olson developed a new commercial steel to serve as a corrosion resistant landing gear material, Ferrium S53, using computational modeling to design and develop the alloy in significantly less time than conventional and empirically designed approaches would take. During the course of said work, competing material requirements were weighted against one another and tradeoffs were made to systematically develop optimal application-specific materials [6].

Several commercially available thermodynamic and kinetic software packages, including Thermo-Calc, TC-PRISMA, DICTRA, which were all from (Thermo-Calc Software AB, Solna, Sweden), Pandat (CompuTherm LLC, Middleton, WI, USA), JMatPro (Sente Software Ltd., Guildford, UK), and FactSage (Thermfact/CRCT, Montreal, Canada, and GTT-Technologies, Aachen, Germany) can predict the thermodynamically stable and metastable phases as well as material properties in ceramic and metal alloys as a function of chemical composition. These phases, along with phase fraction, size, shape, and distribution, in turn, enables estimations to be made surrounding the mechanical properties, and therefore performance, of the material. Alloy design (i.e., selecting the chemical composition and optional heat treatment) can be used to develop materials with a desired set of mechanical properties. Quantitative knowledge of the effects associated with the variation of chemical elements (and their respective alloying content) has on predicted phases and properties can further help optimize material design and can be carried out by developing and applying the elemental impact factor (ψ or EIF, depending on the context) method presented herein. In other words, as an aid to the determination of an alloy’s composition, the ψ is a quantitative way to evaluate the influence a particular alloying element has on any phase at a certain temperature or over a range of temperatures. The same can be achieved with respect to the influence a particular alloying element has on a material property as a function of temperature too.

For example, if a manufactured part is observed to fail due to crack initiations at a known phase (or multiple phases), the ψs for alloying elements in the metallic system can be computed for that phase. Alloying elements with a negative ψ, meaning the addition of that element decreases the amount of the phase under investigation, could be added to the system. This is especially beneficial in cases where undesired phases are caused by the presence of impurity elements that cannot be fully removed from the alloy, but in many cases can be controlled to some degree. Likewise, for the growth of desired phases, elements with a positive ψ could be increased in the system during production to promote the amount of the desired phase and elements with a negative ψ could potentially be decreased, though the addition of elements is typically easier than a reduction in elements in practice, unless the alloy is made from scratch. Finally, the addition of elements not traditionally present in the alloys being used may be examined with the ψ to determine potentially eligible elements that may increase or decrease the amount of desired or deleterious phases of the system. In the case of all of the above examples, the desired as well as undesired phases should first be known in order to find ψ useful. This often requires experimental investigation into the material and its failure mechanisms or consideration of prior literature of relevance. However, once these phases are known, the benefits may be great. As with many computational approaches, the time, resources, and monetary savings from modeling as opposed to experimentation are significant. Of course, models cannot be substituted entirely for experiments; nevertheless, the ψ can be used to determine calculated candidate chemical compositions for alloys to compliment and accelerate the development and discovery of innovative alloys of interest.

Numerous software packages utilize the CALPHAD (“CALculation of PHAse Diagrams”) approach commonly associated with modern thermodynamic modeling [9]. The thermodynamic analysis presented herein utilized Pandat, Thermo-Calc, and JMatPro software packages. While thermodynamic models provided useful equilibrium data, they could not solely be used to predict the complete behavior of a system. As such, kinetic considerations needed to be made since materials are typically not present in equilibrium states. Thermodynamic models predict data that can, however, be used in conjunction with kinetic and solidification models to produce a more accurate prediction. In order to model many kinetic phenomenon, thermodynamic parameters are required. For example, the chemical potential from thermodynamic predictions were used to calculate non-equilibrium diffusion behavior. Similarly, a thermodynamic description of a material system can be used as input into solidification models [10].

JMatPro is the only software package that was utilized during the course of this work that easily considers both thermodynamics and kinetics. Thermo-Calc performs strictly equilibrium calculations, but another software package or module by Thermo-Calc Software is also available and is known as TC-PRISMA, which accounts for kinetics. Thermo-Calc output data can be manually entered as input into the TC-PRISMA software. With the aid of faster processing and greater computational power and capacity, modeling in general has become more common and more easily accessible. This is particularly true for kinetic models of multicomponent systems, especially for alloys with upwards of nine elements. Computing time increases significantly with the addition of each element, resulting in lengthy calculations. Numerous papers have been written that exploit the relatively new capability of kinetic models, particularly with the arguably more user-friendly JMatPro platform [11,12,13,14,15]. Of the kinetic software packages, JMatPro requires the fewest parameters to be input by the user, making it an effective tool when experimental parameters are not available.

With respect to the thermodynamic and kinetic software packages of specific relevance, JMatPro, by Sente Software, uses vast thermodynamic and kinetic databases to calculate stable and metastable equilibrium phases and resulting mechanical, chemical, physical, and thermophysical properties. JMatPro also calculates equilibrium and non-equilibrium solidification and phase transformation behavior. On the other hand, Thermo-Calc, by Thermo-Calc Software, uses the thermodynamic principle of the minimization of Gibbs Free Energy (GFE) to create equilibrium isotherms and isopleths for multicomponent material systems, wherein databases are developed experimentally and even computationally using the CALPHAD approach. As for TC-PRISMA, which is also by Thermo-Calc Software, the thermodynamic databases and calculations of Thermo-Calc are built upon by way of considering diffusion and kinetic factors to simulate concurrent nucleation, growth, and coarsening. This is achieved via the employment of the Langer–Schwartz theory and the Kampmann–Wagner numerical method for multicomponent and multiphase systems. The final platform used herein was Pandat by Computherm LLC, which utilizes the CALPHAD approach to develop multicomponent phase diagrams.

### 1.1. Background Information

The basis on which all of the software packages utilized in this work (Thermo-Calc, JMatPro, Pandat, and TC-PRISMA) is that of the CALPHAD approach. Prior to using the CALPHAD approach, phase diagrams were typically determined experimentally for binary, but occasionally ternary, alloys. While these proved to be relatively accurate, they were hardly useful in “real-world” applications where multicomponent alloys were more commonly used. Multicomponent CALPHAD predictions are based on theoretical and empirically derived data that are extrapolated from binary and ternary systems. Simply put, the CALPHAD approach utilizes the minimization of a system’s GFE to determine a universal equilibrium. Occasionally, software may mistake a local equilibrium for a universal equilibrium. Software parameters can be adjusted to decrease the likelihood of this occurrence, often only at the sacrifice of processing time. In order to calculate the GFE of a system, thermodynamic values of the various binary alloy systems must be determined experimentally.

The calorimetric method is used to experimentally measure many of the necessary parameters used in CALPHAD predictions. Adiabatic calorimeters are used to measure the heat absorbed or emitted by a sample when heated continuously in an adiabatic environment. Heat capacities and resulting enthalpies of transformation can be measured using this method. An additional method for measuring enthalpies of transformation include differential scanning calorimetry (DSC) and differential thermal analysis (DTA). DSC measures the heat absorbed or emitted during a phase transformation or reaction. DTA uses a similar method, but rather than heating at a constant rate, a reference material is used to set the rate, and the difference in heat required to maintain identical temperatures is used to determine the enthalpy [16].

Activities of components are often calculated using gas phase equilibria techniques. They utilize the relationship between the activity of component i, $a_{i}$, with the vapor pressure of component i, $p_{i}$, and the equilibrium vapor pressure of i, $p_{i}^{o}$, such that,

$$a_{i}=\frac{p_{i}}{p_{i}^{o}}.$$

This method is popular due to the experimental simplicity associated with the measurement of vapor pressure. Partial Gibbs energies can be calculated by creating electrochemical cells of binary and ternary systems so one may measure resulting electromotive forces. Databases of these parameters are developed by various software companies and are typically the costliest part of a software package due to the extensive research that is required to build a complete database. These databases allow for the calculation of theoretical phase diagrams for binary, ternary, and higher order multicomponent systems. In order to improve on these calculations, experimental binary and ternary diagrams are created. Calorimetric methods and classical metallographic analysis are typically used for these experiments. Since calorimetric methods might better represent a metastable equilibrium condition, metallography, such as optical and scanning electron microscopy, as well as X-ray diffraction and transmission electron microscopy, are also used. These compiled databases and experiments are one explanation for the variation in outputted results by the various software packages.

The GFE of a system is calculated using the free energy values of each phase. Phases are either solutions or stoichiometric compounds. The two equations for these types are given below, respectively,

$$G=G^{o}+G_{mix}^{ideal}+G_{mix}^{XS},$$

and

$$G_{\left|T,\;\;P\right|}=H_{\left|T,\;\;P\right|}-\left(T\right)\left(S_{\left|T,\;\;P\right|}\right),$$

where $G^{o}$ is the free energy contribution of the pure components of the phase, $G_{mix}^{ideal}$ is the ideal mixing energy contribution, $G_{mix}^{XS}$ is the Gibbs excess energy of mixing (also thought of as the contribution from non-ideal interactions between components), and $H_{\left|T,P\right|}$ as well as $S_{\left|T,P\right|}$ are the enthalpy and entropy as a function of temperature and pressure, respectively. Various models are used by each software package to account for the various types of solutions (i.e., simple mixtures, dilute solutions, ideal solutions, regular solutions, etc.). Standard thermodynamic principles are applied to each situation. More information regarding the specific thermodynamics involved in these calculations may be found in Section 5.3 of the book titled *CALPHAD*—*A Comprehensive Guide*.

Extrapolation of the thermodynamic properties of a binary system is based on the summation of excess parameters utilizing various geometrical weightings of the mole fractions of the components [17]. The excess energy of a multicomponent system, which is utilized by JMatPro (at the very least), is given by the Muggianu equation [18]. The CALPHAD method has a simple goal: to minimize the GFE of the system, which is mathematically represented as G herein. The equally simple rule of mixtures can be applied to calculate this total, where

$$G=\underset{i}{\sum }n_{i}\bar{G_{i}},$$

while $n_{i}$ is the amount of component i and $\bar{G_{i}}$ is the chemical potential of component i, which is represented by the following equation,

$$\bar{G_{i}}=\bar{G_{i}^{o}}+RT\ln a_{i},$$

such that $\bar{G_{i}^{o}}$ is the standard chemical potential. Considering the standard mass balance,

$$\underset{i}{\sum }\alpha_{i,j}x_{i}=n_{j}\forall \;\left(j=1,\;2,\;\ldots m\right),$$

wherein $\alpha_{i,j}$ is the number of atoms of element j in species i, $x_{i}$ is the number of moles in species i, and $n_{j}$ is the total number of moles of j in the system.

That being said, in 1957, Dantzig et al. developed a linear programming approach for the aforementioned mathematical relations, such that,

$$\frac{G}{RT}=\underset{i}{\sum }x_{i}\left(\frac{\bar{G_{i}^{o}}}{RT}\right)+x_{total}\sum^{\;}\left(\frac{x_{i}}{x_{total}}\right)\ln\left(\frac{x_{i}}{x_{total}}\right),$$

while

$$x_{total}=\underset{i}{\sum }x_{i}.$$

Thanks to the analytical approach adopted by Dantzig et al., the GFE of the system may be calculated as a linear function dependent upon

$$\left(\frac{x_{i}}{x_{total}}\right)\ln\left(\frac{x_{i}}{x_{total}}\right).$$

The minimal value for G is therefore computed in an iterative fashion, which reveals the theoretical framework underpinning the basis for each of the thermodynamic software platforms utilized during the course of this research effort. In fact, the linear programming approach calculates a point equilibrium for a defined composition, temperature, system size, and pressure, with no degrees of freedom available. It is often more useful to determine the equilibrium state of a system varying one or two of those properties, thus resulting in a line or map calculation, respectively. A map calculation is more commonly referred to as a phase diagram or isopleth. A step diagram changes one property incrementally from a minimum to a maximum and calculates the free energy at each step.

In the phase diagrams, the temperature and composition (or pressure and composition) are approached as a series of step calculations, which are in turn a series of point calculations. Phase diagrams are calculated by sequentially incrementing the temperature (or pressure) as well as the composition and recognizing boundaries where one phase varies from one side of the boundary to the other. Each commercially available software package has its own proprietary method for determining the boundary conditions within a user-defined tolerance. In an additional attempt at improving calculations, the developers of said software packages have incorporated extensive experimental data for binary and ternary systems. Each package also has its own error minimization algorithms that are used to combine experimental data with theoretical data. Two of the most common optimization programs are “PARROT” and “Lukas” and make up the brains, so to speak, of two of the most common commercially available CALPHAD-based software packages, Thermo-Calc and JMatPro, respectively.

Kinetic additions to CALPHAD software packages were initially added by manually suspending stable phases to leave metastable phases. Since then, improvements have been made to utilize diffusion, the enthalpy of formation, and other parameters to make more robust models. Gulliver 1922 and Scheil 1942 models are utilized in commercially available software’s of relevance and have proven to be extremely accurate for conventional cooling rates when compared to experimental values, particularly for aluminum alloys, which are considered during this proof-of-concept study. The Scheil model assumes that solute diffusion in the solid phases is negligible and diffusion in the liquid is fast enough for one to assume complete diffusion. Since kinetic considerations for micro-segregation and back-diffusion are not considered, this is actually a truly kinetic model. As highlighted above, the software package by Thermo-Calc Software, TC-PRISMA, is based on the Langer–Schwartz theory and the Kampmann–Wagner numerical method. This allows for the simultaneous solution of concurrent nucleation, growth, and coarsening of dispersed phases.

JMatPro software also incorporates a material property database to predict material properties of the alloys for point and step calculations for stable and metastable phases. Properties have been measured experimentally for various precipitates in various material types (i.e., aluminum alloys, titanium alloys, steels, etc.) for precipitates of various shapes and morphologies. These data include the molar volume, the thermal conductivity, the Young’s modulus, and the Poisson’s ratio, which are calculated using basic pair-wise models for multicomponent systems. While specific details of the algorithms used are proprietary and maintained by the developers of JMatPro, they are similar to the thermodynamic excess functions used in multicomponent alloys. Once the individual phase properties are defined, these data are called upon by the software to predict properties of multicomponent alloys using proprietary mixture models. Extensive experimental verification has been reported in [12]. Additional material properties can be calculated using known relationships between certain properties (e.g., electrical and thermal conductivity) without the need for additional databases. Other properties that this pertains to includes volume, density and expansion coefficients; Young’s, bulk, and shear moduli; the Poisson’s ratio, thermal conductivity, and diffusivity; and electrical conductivity and resistivity, among others.

Examples of theoretical relations between material properties that were not utilized in this particular work, but remain viable, include the calculation of tensile strength and hardness from the Hall–Petch relationship, consideration of grain coarsening by Ostwald Ripening, and Jominy Hardenability in steels by continuous-cooling transformation (CCT) curve calculations. Time–temperature–transformation (TTT) and CCT diagrams are also calculated in JMatPro using the Johnson–Mehl–Avrami equation as a foundation. Since the original work was for spherical precipitates, additional changes have been applied by developers to account for the effect of non-spherical precipitates. Not only are the additional model equations needed, work also needs to be done to assess the nucleation shape and characteristics for each precipitate along with experimental comparison. Strengthening mechanisms by the various phases present are incorporated by solid solution strengthening as well as precipitation hardening. If the material is not recognized, semi-empirical approaches are used instead of the physical models, as highlighted in [19].

### 1.2. On the Selection of Alloyed Aluminum

Material properties are directly dependent on their microstructure. One way to alter microstructures, and therefore material properties, is through the use of thermal treatments. Heat-treatable alloys exploit thermal treatments to improve their properties, typically involving a series of steps: homogenization, annealing, solution heat treatment, quenching, natural aging, and artificial aging. The alloys considered here are heat-treatable: Al 2024 (Al–Cu alloy), Al 6061 (Al–Mg–Si alloy), and Al 7075 (Al–Zn alloy).

The purpose of the homogenization step is to allow the alloying elements to form a homogenous solid solution within the Al matrix. This should remove any micro-segregation and allow for evenly dispersed precipitates to form during subsequent thermal processing steps. Homogenization is performed in the 350–450 °C temperature range for Al alloys and may often take 15–20 h, depending on the size of the part. The annealing step is used to relieve residual stresses induced during forming by softening the material; the goal of this is to make the material easier to work with during subsequent forming processes. Annealing is performed at temperatures above the recrystallization temperature, in the 350–450 °C range, and may take three to four hours depending on the size of the part and the amount of strain present in the material.

The solution heat treatment step has a goal similar to homogenization—to form a uniform solid solution—though the solution step brings the alloy closer to completion of this goal than that of homogenization. Solution treatments are carried out at higher temperatures than homogenization treatments, in the 450–550 °C range, which is closer to the eutectic melting temperature of the alloy. Local variations in solute concentration may exist in the material during any of the steps, but it is most important in the solution thermal processing step. These local variations may cause a change in the eutectic temperature, which cause some localized, incipient melting. Incipient melting causes a decrease in properties but can be avoided through careful selection of treatment temperature and control of the heating rate. The time held at a temperature is very important, but also extremely variable depending on part dimensions. For example, thin sheets may require a matter of minutes while thicker castings may take over 20 h. If the time at temperature is too short, a full solid solution will not be achieved, but if it is too long, high temperature oxidation becomes a risk. After the solution treatment, the part is quenched to retain the supersaturated solid solution; this retention achieves optimal precipitation hardening in subsequent steps. The quench rate is of high importance; it must be fast enough to avoid precipitation but slow enough to avoid distortion.

The purpose of the aging steps is to nucleate and grow small coherent or semi-coherent precipitates and their precursors, i.e., Guinier–Preston (GP) zones, for example. First, solute atoms cluster around quenched-in vacancies; their lattice mismatch causes strain fields to form, increasing the strength of the material. Once sufficient solute atoms have diffused to the cluster, coherent precipitates can form, increasing the strain in the lattice. As more atoms diffuse to the precipitate, the precipitate continues to grow and increase the lattice strain, until the matrix can no longer accommodate the amount of strain. At this point, the precipitate loses some coherency and becomes semi-coherent. As more atoms continue to diffuse, the precipitate continues to grow, increase strain, and lose coherency, until it becomes incoherent in its equilibrium form. The precursor phases provide the most increases in strength, due to the large amounts of strain in the matrix. The natural aging step is used to strengthen alloys at room temperature, though not all alloys are capable of this. For example, the aluminum-copper alloys, which are commonly expressed as the 2xxx series, harden substantially at room temperature over a few days and their properties become stable after 1 week. On the other hand, the aluminum–magnesium–silicon alloys, also known as the 6xxx series, and the aluminum-zinc-magnesium-copper alloys, also known as the 7xxx series, may harden at room temperature, but their properties may still change even after a few years at room temperature. Artificial aging may be used in situations like these to accelerate the process of achieving stable precipitates. Artificial aging uses elevated temperatures, in the 100–200 °C range, to accelerate the processes that would occur naturally at standard conditions.

The predominant precipitate in the 2xxx alloy series is Al_2_Cu (θ), with its precursors ″ and θ′. In 6xxx series alloys, Mg_2_Si (β) is the equilibrium phase, with precursors β′ and β″. The 7xxx series have several precipitation sequences that may occur, depending on the exact alloy composition and local concentrations. Al_2_CuMg (S- phase), Mg_32_(Al, Zn)_49_ (T phase), and MgZn_2_ (η) may be the predominant equilibrium phases with precursors T′ and η′.

### 1.3. Motivation and Objectives

With the information provided for alloyed aluminum systems, the motivation and objectives of this study were formulated and presented next. Since the original public release and publication of the MGI report already mentioned herein, numerable subsequent publications attempting to incorporate the integrated computational materials (ICME) approach to materials research and design. Examples of such studies were introduced by a guest editor for a special issue of *JOM: The Member Journal of The Minerals, Metals & Materials Society* in an article titled “CALPHAD-Based Integrated Computational Materials Engineering Research for Materials Genomic Design” by Xiong in [20]. As part of the special issue, Bhat et al. proposed a rules-based approach to database and simulation tool development; Chen et al. looked at multidimensional phase diagrams; and Zhu et al. published a paper titled “Molar Volume Modeling of Ti-Al-Nb and Ti-Al-Mo Ternary Systems”. While research is clearly underway surrounding the MGI, such as CALPHAD and ICME integration, the present manuscript was also motivated by the fact that the ψ can be used to determine calculated candidate chemical compositions for alloys to compliment and accelerate an alloy’s development and discovery for mesoscale integration into the ICME framework.

## 2. Methods and Computational Thermodynamic and Kinetic Models

Thermodynamic calculations were performed using Thermo-Calc, version 4.1, with the TCAL3 database. Metastable kinetic phase predictions were calculated with TC-PRISMA, version 2.0.2, using the TCAL3 and MOBAL3 databases, wherein both of which were version 3. The properties calculations were made in JMatPro, version 8, using their magnesium and aluminum databases. Calculation of equilibrium phase fractions utilized the average (or experimentally measured) composition within Al 6061’s military-grade specification. Each alloying elements content was varied from, at least, −99 to +99 wt.% of the average compositional value associated with a given alloy, divided by an integer to get the incremental compositional change. For example, take the average compositional content in wt.% associated with the alloying element chromium in Al 6061, which is 0.195 wt.%. As a result, the −99 wt.% minimal boundary condition is to be interpreted such that the initial elements content would be the average value less the quantity of 99% of the average value. Accordingly, the −99 wt.% boundary value would yield a lower limit of 0.002 wt.% wherein the difference between 0.002 and 0.195 wt.%, 0.193 wt.%, would be added to the aluminum matrix content as part of the balance. One may now easily appreciate the opposite case concerned with +99 wt.% rather than −99 wt.%. One element composition was varied at a time, while the remaining elements were left at their average designated/specified value, or an experimentally derived value. The equilibrium phase fractions were calculated for each composition as a function of temperature. The temperature range for every calculation was between 0 and 700 °C, with a maximum temperature step of 10 °C. Since Al 6061 was selected for careful inspection and proof-of-concept analysis of the ψ, the compositional ranges and alloying elements associated with this heat-treatable light alloy are given below.

The aluminum alloy known as Al 6061 is widely used for a range of applications and was therefore used in the ψ analysis for stable phase and property predictions in this study. This kept with the MGI’s focus on the use of modeling to develop “lightweight protection materials”. Al 6061’s average specification for composition in wt.% is: 0.195 Cr, 0.275 Cu, 0.35 Fe, 1.0 Mg, 0.075 Mn, 0.6 Si, 0.075 Ti, 0.125 Zn, and 97.3 Al, according to [21]. As a result, this was the baseline composition generally utilized by the authors and are referred to when relative variation in wt.%s are discussed hereafter. The metastable data were calculated using the same compositional increments associated with the inspection of stable phases, though temperature values were held constant, observing the temporal evolution of precipitate amount and size.

Recall the fact that Thermo-Calc was used to generate the thermodynamic data as a function of temperature and alloy composition. Thermo-Calc’s TCAL3 aluminum database, version 3, was used herein. Both the software and database have been experimentally validated in numerous experimental tests [22,23,24,25,26,27]. While this paper does not detail said results, they are readily available in the aforementioned references. Additionally, this work utilized JMatPro software to calculate thermophysical, physical, and mechanical properties as a function of alloy composition and temperature. Examples of these properties include yield and ultimate tensile strengths, electrical and thermal conductivities, viscosities, among others. Experimental results validating these JMatPro models can be found in numerous papers too, a few of which are listed here [28,29,30].

**Figure 1:**
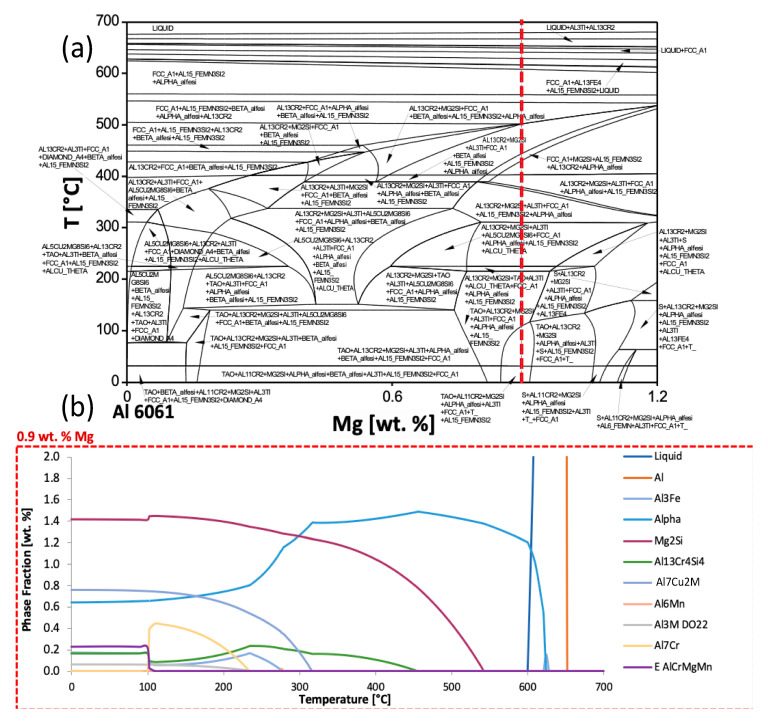
The temperature versus magnesium wt.% in the Al 6061 system considered is presented in (**a**) and represents an isopleth of the composition created using Pandat wherein each thermodynamically predicted equilibrium phase in the isopleth was labeled for emphasis and demonstrative purposes. The vertically dashed red line represents a magnesium wt.% of 0.9. From the isopleth in (**a**), the equilibrium phase fractions in Al 6061 at 0.9 wt.% magnesium as a function of temperature for the composition associated with the red line from (**a**) is presented in (**b**).

The impact of chemical composition on alloy phase transformation kinetics are also discussed. Specifically, the effects of time, temperature, and composition on the phase fraction and diameter of precipitate phases are investigated. For the kinetic models, Al-Cu-Mg and Al-Cu-Mn systems were studied, with specific analysis on the stable precipitate theta (Al_2_Cu). Nevertheless, to initialize this research, efforts to predict phase equilibria of the Al 6061 composition considered, computational thermodynamic principles were used to develop equilibrium isopleths and isotherms. Isopleths show phases present under equilibrium conditions. The minimization of GFE was performed to yield the most thermodynamically stable phases. Both Pandat and Thermo-Calc Software packages were used for these calculations. An isopleth of Al 6061 is shown in Figure 1. To demonstrate the numerous equilibrium phases, which are present as a function of magnesium content and temperature, the phase names were assigned to the isopleth in Figure 1a. Recall that this paper primarily presents the outputs for Al 6061 as an example of the types of modeling and analysis performed during the course of this work; however, the concepts, methods and approach may be readily extended to a vast array of alternative alloys alongside compositions of interest.

In Figure 1a, the red dashed vertical line at 0.9 wt.% magnesium represents a suitable magnesium content within the allowable range prescribed for Al 6061 as defined by military-grade specifications, which is between 0.8 wt.% and 1.2 wt.%. If one were to imagine comparable vertical dashed lines at 0.8 wt.% and 0.9 wt.%, several different phases would be present under equilibrium conditions depending on the composition of the particular batch of Al 6061 alloys procured despite the fact that an alloy shares a commonly specified compositional range. Therefore, Figure 1a serves as an example of how these diagrams may be used to visually appreciate and capture how the alteration of chemical composition of an element can produce desired or deleterious secondary phases, and if the alloy exhibits the potential for precipitation hardening or thermal processing.

**Figure 2:**
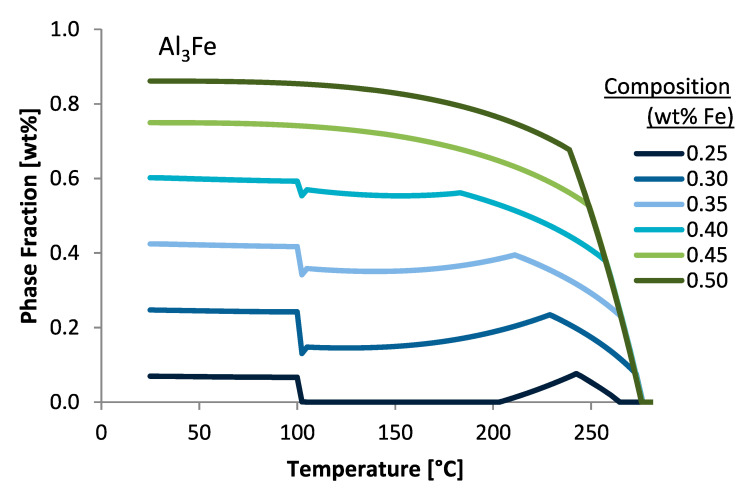
Variation of the predicted equilibrium for the Al_3_Fe phase in Al 6061 with a content variation of the alloying element iron, as a function of temperature, between room temperature and more than 250 °C.

As for Figure 1b, similar thermodynamic principles were used to render the equilibrium phase fractions for Al 6061 at 0.9 wt.% magnesium content as a function of temperature. Face-centered cubic (FCC) aluminum was the primary phase, though it is out of viewing range over the majority of the temperature range depicted due to the scale of the diagram. The major secondary intermetallic phases predicted were Mg_2_Si, Al_7_Cu_2_Fe, and alpha, which is also expressed as Al_47_(Fe,Mn,Cr)_11_Si_5_. As will subsequently be considered and discussed in greater detail throughout the next section of this original research article, as well as the remainder of the manuscript, phase variation plots, via computational thermodynamic software-based inspection, can assist in observing the effect of varying alloying element and content on phase stability as a function of temperature. Such a mode of analysis also utilizes thermodynamic principles for theoretical observation. Figure 2 illustrates this mode of analysis by way of plotting the Al_3_Fe phase fractions predicted under equilibrium conditions as a function of temperature for varied iron wt.% compositions.

**Figure 3:**
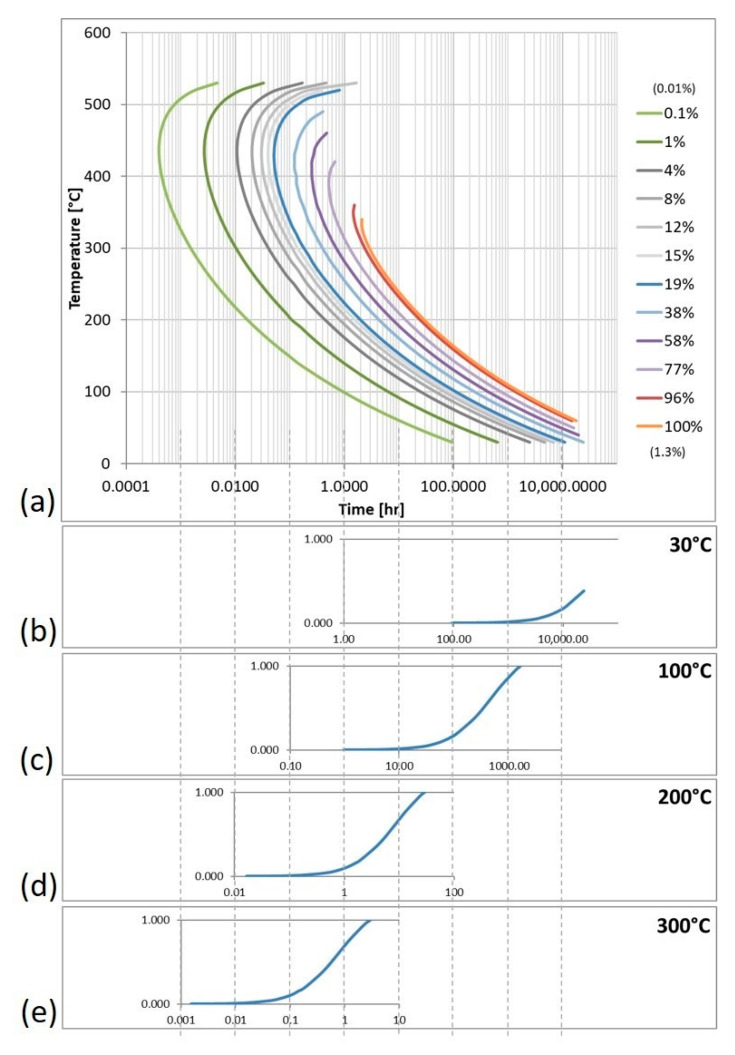
(**a**) The TTT diagram for the Mg_2_Si phase in Al 6061, thus showing the amount of Mg_2_Si formed as a function of time and temperature, plotted on a semi-log chart. (**b**–**e**) The fraction of Mg_2_Si transformed as a function of time for various temperatures.

As was just discussed, the thermodynamic models utilized above were all derived from the minimization of the GFE of a given system. Accordingly, such an approach is only valid for equilibrium conditions. While equilibrium is a fair assumption to make in order to acquire an approximate solution, in order to obtain a more accurate sense of a systems behavior, kinetic consideration must also be given. Having presented Figure 1a, another isopleth of Al 6061 was not presented in Figure 3. That being said, recall the fact that the red vertical line at 0.9 wt.% magnesium in Figure 1a represents a suitable magnesium content within the allowable and standardized range prescribed for Al 6061, which is between 0.8 wt.% and 1.2 wt.%.

Considering the above assertion that one could imagine comparable vertical dashed lines at 0.8 wt.% and 0.9 wt.%, several different phases would be present under equilibrium conditions depending on the composition of the particular batch of Al 6061 alloys procured despite the fact that an alloy shares a common composition within a specified range. Nevertheless, Figure 1b can be contrasted to Figure 3a, which considers kinetic effects, such as diffusion behavior, to more accurately predict phases present in a system as a function of time. Figure 3a is a TTT diagram for Mg_2_Si in Al 6061 that was created using data derived and computed by way of employing JMatPro. These data can be further manipulated to create plots showing the fraction of Mg_2_Si that has formed as a function of time at various temperatures, as shown in Figure 3b–e.

An example of the type of data that can be extracted from these types of predictions compared results from Figure 1b and Figure 3a. For 200 °C, and at equilibrium in Figure 1b, there is 1.39 wt.% of Mg_2_Si. However, from the TTT diagram shown in Figure 3a, at 200 °C, it takes 1.0 min for Mg_2_Si to nucleate and form. At room temperature, the difference is more exaggerated, and it takes nearly four days for Mg_2_Si to begin to form. It is evident that temperature plays a large role in the type and quantity of phases present. In fact, the time it will take for 100% of the possible Mg_2_Si phase varied by 1641%. The simulations used to create these kinetic diagrams did not run to times long enough to reach 100% Mg_2_Si at low temperatures. In fact, data for higher temperatures were fit to a power curve to generate an equation from which the approximate time to reach complete Mg_2_Si formation at room temperature was calculated to be 602 years. This result is not at all evident when observing only the equilibrium isopleth and shows the necessity of kinetic considerations.

## 3. The Elemental Impact Factor Applied to Phase Stability

**Figure 4:**
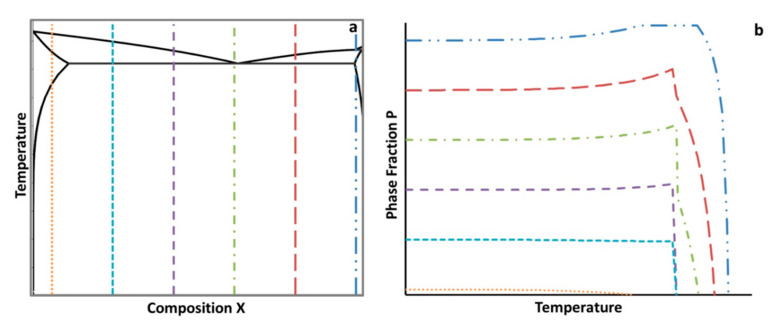
Schematic setup for the ψ. (**a**) A schematic of an isopleth with varying temperature and composition of element X. The vertical lines represent contents of a hypothetically varied element, which was assigned the label X. (**b**) A schematic of the amounts of the imaginary equilibrium phase P predicted as a function of temperature with the corresponding composition change of X from (**a**).

A schematic depicting the concept of the ψ for material phases is given in Figure 4. A sketch of an isopleth is therefore shown in Figure 4a. The vertical dashed lines represent a variation in a hypothetical alloying element, which was labeled X. Figure 4b represents the resulting amount of a hypothetical, arbitrarily labeled, phase P as a function of temperature, which was calculated from a lever rule type analysis from the equilibrium isopleth. In practice, both types of diagrams for specific alloy systems can be computed and created using thermodynamic software. While the two schematics given in Figure 4 are not representative of a specific alloy system and composition, the isopleth shown in Figure 4a is somewhat comparable in shape and form with that of a castable Al–Cu binary alloy, which is consistent with the focus upon an aluminum alloy throughout the course of this work, as detailed in the Methods and Computational Thermodynamic and Kinetic Methods section of the present manuscript.

That being said, in an effort to quantify the effects that chemical elements have on the presence of equilibrium phases, non-equilibrium phases, and material properties, the ψ was developed. The ψ estimates the influence that a change in the content of a particular chemical element has on a phase fraction, precipitate size, or material property at a specific temperature. Time must also be specified for metastable calculations. To determine the ψ for a phase fraction, thermodynamic or kinetic software can be utilized to assess the thermodynamically stable or metastable phase fractions. In theory, Figure 4b could be quantified for a distinct temperature using the ψ, which is given by

$$\psi_{element,\;T}^{phase}=\frac{\Delta \mathrm{wt}.\%\;Phase}{\Delta \mathrm{wt}.\%\;Element}=\frac{P\left(X_{j}\right)-P\left(X_{i}\right)\;\left[\mathrm{wt}.\%\right]}{X_{j}-X_{i}\;\left[\mathrm{wt}.\%\right]},$$

where *P(X_i_)* is the equilibrium or non-equilibrium phase fraction for *P* at a compositional wt.% for *X* where *i* is the lowest compositional increment (or a relatively low incremental compositional change between the lowest compositional increment and a larger composition increment, and vice a versa for *j*) of the hypothetical element *X* and *j* is the highest compositional increment at a specified temperature, *T*, such that i≠j.

Nevertheless, while utilizing or applying the EIF method/approach to assess the compositional influences on phase stability, for example, within a given alloy system, one must remain aware of the fact that the EIF depends upon the alloying chemistry, the number of incremental compositional steps computationally considered and calculated, and the start/end points defined. Furthermore, in so far as the EIF has been defined herein, one must also keep in mind the fact that the change in alloying elemental content for a given constituent within an alloy is accounted for by way of varying the base or matrix materials content. More specifically, when a given alloying element is increased within the system, the base material is proportionally decreased and vice a versa. Since alloyed aluminum systems were explored herein for the purpose of demonstrating the veracity of the EIF for performance-driven alloyed material design, a decrease/increase in an alloying element would be accounted for by an increase/decrease in the amount of aluminum present accordingly. Finally, as will be discussed in Section 7 of the present manuscript, changes in phase equilibria as well as the occurrence of phase transformations can influence EIF-based analysis through the (Δwt.% Phase) numerator of the ψ expressed above.

**Figure 5:**
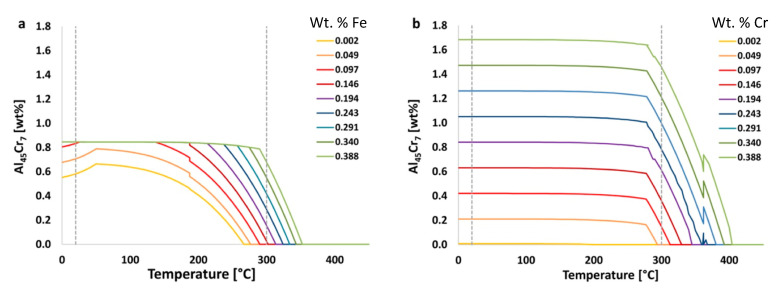
Example used to demonstrate the input for calculating ψs. (**a**) The variation of the calculated equilibrium Al_45_Cr_7_ phase in Al 6061 with iron variability. (**b**) The variation of the calculated equilibrium Al_45_Cr_7_ phase in Al 6061 with a content variation of chromium. The temperature of 20 °C is represented by the left-most dashed vertical line. The temperature of 300 °C is represented by the right-most vertically dashed line imposed upon the graphs.

In practice, Figure 5 shows the effects that the amount of iron and chromium have on equilibrium amounts of the stable Al_45_Cr_7_ phase. In Figure 5b, it can be seen visually and qualitatively that chromium has a greater effect on the resultant amount of Al_45_Cr_7_ phase than does iron. This, of course, makes intuitive sense given the fact that Al_45_Cr_7_ incorporates chromium as an element within the inorganic intermetallic precipitate and does not directly incorporate iron as such.

For example, using data shown in Figure 5a with the equation just provided for the EIF yields the following for the effect of iron on the Al_45_Cr_7_ phase at 20 °C,

$$\psi_{Fe,\;\;\;20\;{}^{\circ}C}^{Al_{45}Cr_{7}}=\frac{P\left(X_{1}\right)-P\left(X_{2}\right)\;\left[\mathrm{wt}.\%\right]}{X_{1}-X_{2}\;\left[\mathrm{wt}.\%\right]}=\frac{0.846-0.581}{0.388-0.002}=\;0.7,$$

where the ψ should be used as a relative value to be compared with other ψs for various elements, phases, temperatures and times.

Calculated in the same way, but for the element chromium, the ψ value associated the effect of chromium on Al_45_Cr_7_ at 20 °C, or $\psi_{Cr,\;20\;{}^{\circ}C}^{Al_{45}Cr_{7}}$, is 4.3. Comparatively speaking, the content of chromium has about six times the impact on the amount of Al_45_Cr_7_ present at 20 °C than that of iron. While this is to be expected, since chromium is present in this particular phase, it is not as intuitive, for non-experienced or general materials scientists, that iron, which is not in this phase, also has a significant and non-negligible influence on the amount of the Al_45_Cr_7_ phase present. This is an example of how the use of the ψ in other situations may lead to unexpected relationships between chemical composition and phases, which may otherwise go unnoticed. Thus, ψ-based analysis allows for rapid or high-throughput correlation between elements and their influence on phase fractions present, regardless of whether or not a given element maintains explicit inclusion as a constituent, substitutional or otherwise, within a given phase.

**Figure 6:**
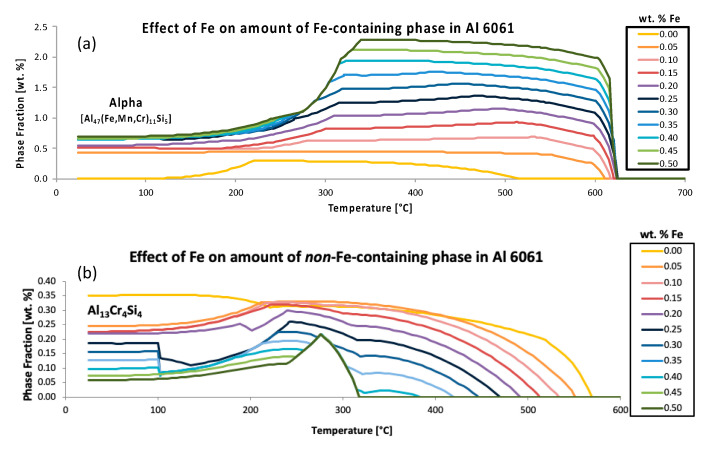
(**a**) The variation of the predicted equilibrium alpha phase in Al 6061 with a compositional variation of iron as a function of temperature. The graphical rendering, shown in (**a**), of alpha phase fractions as a function of temperature, for a range of iron contents, enabled the exploration of the effect of iron on the amount of an iron-containing phase in Al 6061. Alternatively, (**b**) is similar to (**a**); however, (**b**) concerns the predicted equilibrium Al_13_Cr_4_Si_4_ phase in Al 6061 with a content variation of iron as a function of temperature. Consequently, the graphical rendering presented in (**b**) of the Al_13_Cr_4_Si_4_ phase fractions as a function of temperature for a range of iron contents also enabled the exploration of the effect of iron on the amount of a non-iron-containing phase in an Al 6061 system.

Accordingly, Figure 6 aims to not only compliment Figure 5, but to also present two additional inspections of the effect of iron, in Al 6061, on another iron-free phase other than Al_45_Cr_7_, namely Al_13_Cr_4_Si_4_, as well as the effect of iron on the iron-containing phase referred to as alpha. Such analysis is consistent with ψ-inspired inspection and the quantitative assessment method. More specifically, Figure 6a presents the variation of the predicted equilibrium alpha phase in Al 6061 with a content variation of iron as a function of temperature. Interestingly, the graphical rendering of the alpha phase fractions, as a function of temperature for a range of iron contents, as shown in Figure 6a, enabled the exploration of the effect of iron on the amount of an iron-containing phase in Al 6061. Alternatively, the Figure 6b plot is similar to the Figure 6a plot; however, Figure 6b concerns the predicted equilibrium Al_13_Cr_4_Si_4_ phase in Al 6061 with a compositional variation of iron as a function of temperature. Consequently, Figure 6b presents the Al_13_Cr_4_Si_4_ phase fractions as a function of temperature for a range of iron contents, thus also enabling the exploration of the effect of iron on the amount of a non-iron-containing phase in an Al 6061 system.

**Figure 7:**
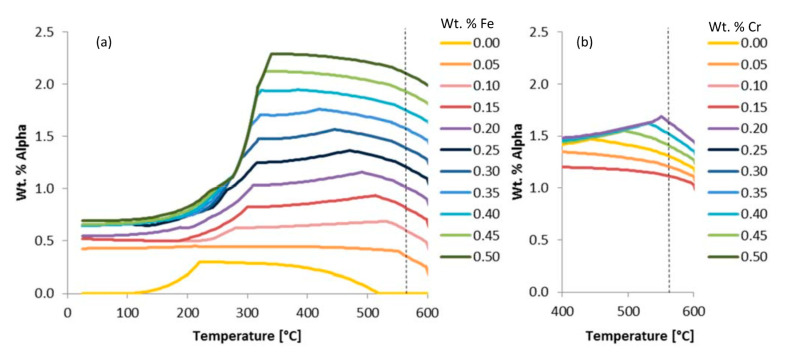
Variation of predicted equilibrium alpha phase in Al 6061 with a content variation of Fe, as shown in (**a**), and a content variation of Cr, as shown in (**b**). The temperature of 575 °C is represented by the dashed vertical lines in (**a**,**b**).

Another pair of examples that invokes the use of the ψ are given in Figure 7. Just as alpha was considered in Figure 6 for the purpose of assessing the chemical or elemental effects of iron on an iron-containing phase known as alpha, Figure 7a does the same, whereas Figure 7b looks at the effect of chromium on the chromium-containing phase alpha, which compliments the robustness of the consideration of ψ herein. This is achieved by way of considering the fact that iron and chromium are both apparently stoichiometrically interchangeable with one another in alpha, but iron was found to maintain a greater influence (i.e., greater ψ) at the same temperature and wt.% compared with the chromium-based comparisons associated with Figure 7. More to the point, the chemical or elemental effect of iron on alpha at 575 °C was deduced according to

$$\psi_{Fe,\;575\;{}^{\circ}C}^{\alpha }=\frac{\Delta \mathrm{wt}.\%\;Phase}{\Delta \mathrm{wt}.\%\;Element}=\frac{2.07-0.00}{0.50-0.00}=4.10,$$

that the ψ value was 4.10 under said conditions. However, calculated in the same way, the ψ associated with the effect of chromium content on alpha at 575 °C was found to be 1.9. Comparatively speaking, iron has about double the effect on the alpha phase present at 575 °C.

**Figure 8:**
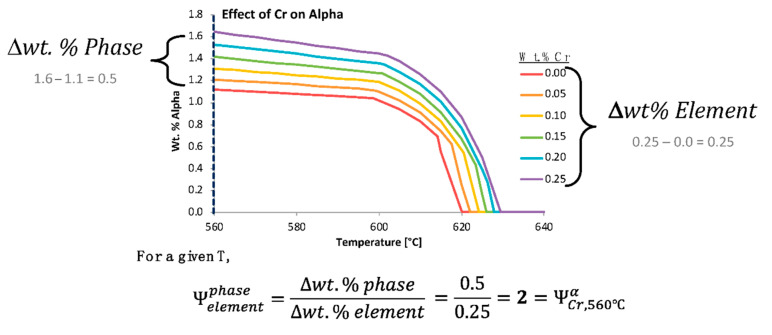
Variation in the prediction of the equilibrium alpha phase in Al 6061 with a content variation of chromium according to the legend provided. The vertically dashed line superimposed upon the wt.% alpha versus temperature curves was included to signify a temperature of 560 °C that was associated with the embedded demonstration of ψ computation.

**Figure 9:**
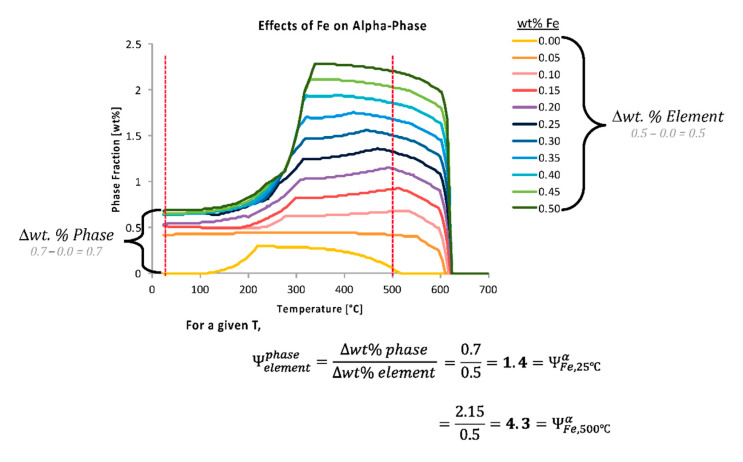
Variation of the prediction of the equilibrium alpha phase in Al 6061 with a content variation of iron according to the legend provided. Two vertically dashed lines were superimposed upon the phase fraction versus temperature curves at 25 and 500 °C, respectively, to identify the temperatures associated with the embedded demonstration of two ψs.

In many cases, the initial wt.% of a given phase may not be zero, which is exemplified below. Stated otherwise, Figure 8 presents the variation of the prediction of the equilibrium alpha phase in Al 6061 with a content variation of chromium according to the legend provided. The vertically dashed line superimposed upon the wt.% alpha versus temperature curves was included to signify the temperature (560 °C) associated with the embedded demonstration of the ψ computations. Additionally, Figure 9 captures the variation in the prediction of the equilibrium alpha phase in Al 6061 with a content variation of iron according to the legend provided. Two vertically dashed lines were superimposed upon the phase fraction versus temperature curves at 25 and 500 °C to identify the temperatures associated with the embedded demonstration of two ψs.

To generate the data required to perform this analysis, thermodynamic and software was used to calculate the equilibrium and non-equilibrium phase fractions, sizes, and properties at selected temperatures and compositions. As the composition ranges selected may alter the results, it is important to identify the ranges for each analysis dependent on any compositional constraints. For the work shown here, the effects of altering elements pre-existing in an alloy are studied; therefore, elemental compositions between −99 wt.% and +99 wt.% were used, as discussed earlier. Using 0 wt.% of the element was obviously not recommended for the present study because of the fact that entirely removing an element often results in the formation of new phases than those being studied for a specified nominal alloying chemistry, as was the overarching case during the course of this work for Al 6061, as already detailed.

**Figure 10:**
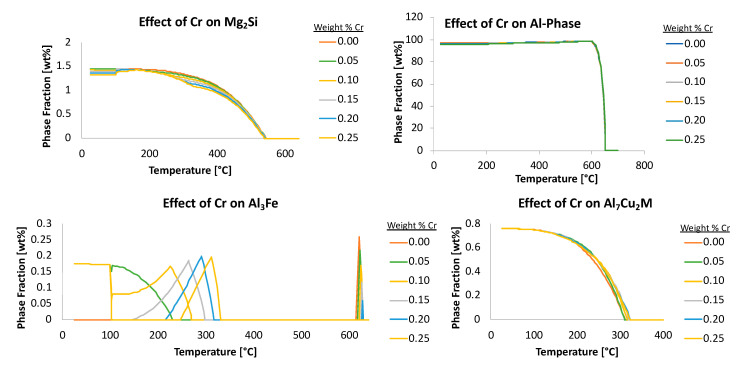
The effect of varied chromium content on the phase fraction of the Mg_2_Si, the aluminum matrix phase, the Al_3_Fe, and the Al_7_Cu_2_M, labeled accordingly, as a function of equilibrium temperatures. The wt.% Cr values corresponding to each curve in the respective plots was color coded in accordance with the respective legends provided.

The compositional range (from −99 to +99 wt.%) for each element was divided into discrete values; thus, becoming the compositional steps. More or fewer content steps can be used, but more steps require longer computational analysis times while fewer steps may lead to less accurate results. Nine iterations were found to balance computational time and accuracy for demonstration and practical purposes using a regular computing source. It should be noted that the unit expressed as weight percent of both element and phase was chosen in this study, but atom and mole percent are equally valid. The TCAL3 thermodynamic database, in conjunction with Thermo-Calc software was used to generate the equilibrium data. One elemental composition was varied at a time, with the average elemental composition used for the remaining elements. Further, Figure 10 captures additional examples of the effects of chromium on four different phases too.

## 4. Property, Kinetic, and Metastable Elemental Impact Factors

**Figure 11:**
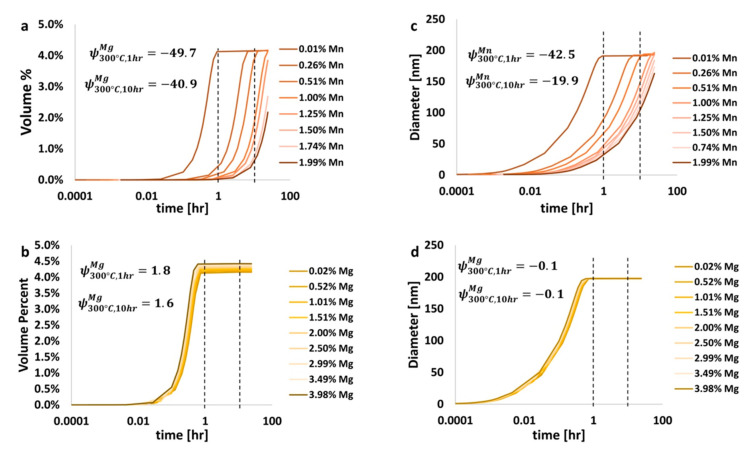
ψ calculations of temporal precipitation properties. At 300 °C, the volume fraction, represented in (**a**,**b**), and precipitate diameter, represented in (**c**,**d**) are calculated for theta in Al-Cu-Mn, as shown in (**a**,**c**), as well as Al-Cu-Mg, as shown in (**b**,**d**).

To calculate the ψ of properties,

$$\psi_{Element,T}^{Property}=\frac{\%\Delta \;Property}{\Delta \mathrm{wt}.\%\;Element}=\frac{\frac{\rho \left(X_{j}\right)-\rho \left(X_{i}\right)}{\rho \left(X_{i}\right)\;}*100\;\left[\mathrm{wt}.\%\right]}{X_{j}-X_{i}\;\left[\mathrm{wt}.\%\right]},$$

such that ρ*(X_i_)* is the magnitude of the property ρ at composition *X*, while *i* is the lowest compositional increment (or a relatively low incremental compositional change between the lowest compositional increment and a larger composition increment, and vice a versa for *j*) and *j* is the highest compositional increment of the element under investigation, *X*. The primary difference between this expression and the equation for the phase fraction EIF is in the numerator of the equation. Examples of data used to generate property ψs are shown in Figure 11. The final type of ψ is that associated with calculating the kinetic precipitate size. This value is not only a function of composition and temperature, but also of time. The equation for this temporal value is

(1) $$\psi_{element,\;T,t}^{diameter}=\frac{\%\Delta \;diameter}{\Delta \mathrm{wt}.\%\;Element}=\frac{\frac{d\left(X_{j}\right)-d\left(X_{i}\right)}{d\left(X_{i}\right)}*100\;\left[\mathrm{wt}.\%\right]}{X_{j}-X_{i}\;\left[\mathrm{wt}.\%\right]},$$

where the numerator represents the percent change in precipitate diameter at a specific time, *t*, and temperature, *T*, between compositions *X_i_* and *X_j_*.

The kinetic software, identified as TC-PRISMA, was used to calculate the metastable phase fractions and precipitate diameters. Note that the software also produces the number of nuclei, the nucleation and coarsening rates, the critical radii, and the precipitate size distribution; all of which can be analyzed using ψs by way of simply replacing the diameters, with any of the aforementioned, in the appropriate mathematical expression. The theta phase, expressed as Al_2_Cu, is an important strengthening phase in Al-Cu-based alloys, such as those in the 2xxx series. The effects of magnesium versus manganese on the amount of and size of theta were analyzed using the ψ. Analysis of the volume fraction of and size of this precipitate as a function of time, temperature, and composition, was performed. One ψ equation was used to calculate the volume fraction ψ while another was used to calculate the diameter ψ. For the purpose of demonstration, consideration of the Al 6061 system defined and discussed thus far was briefly suspended in favor of a simple ternary aluminum alloy with 4.00 wt.% Cu and 0.02 to 3.98 wt.% Mg and/or 0.01 to 1.99 wt.% Mn. Simulations were performed at 300 °C for up to 24 h. Results are shown in Figure 11. Such a brief transition away from military-grade Al 6061 as the system being evaluated through the lens of EIF analysis provided the present authors with the ability to demonstrate the scope and utility of the ψ beyond the single case of Al 6061 and subsequently present said scope and veracity associated with the EIF quantification herein.

**Figure 12:**
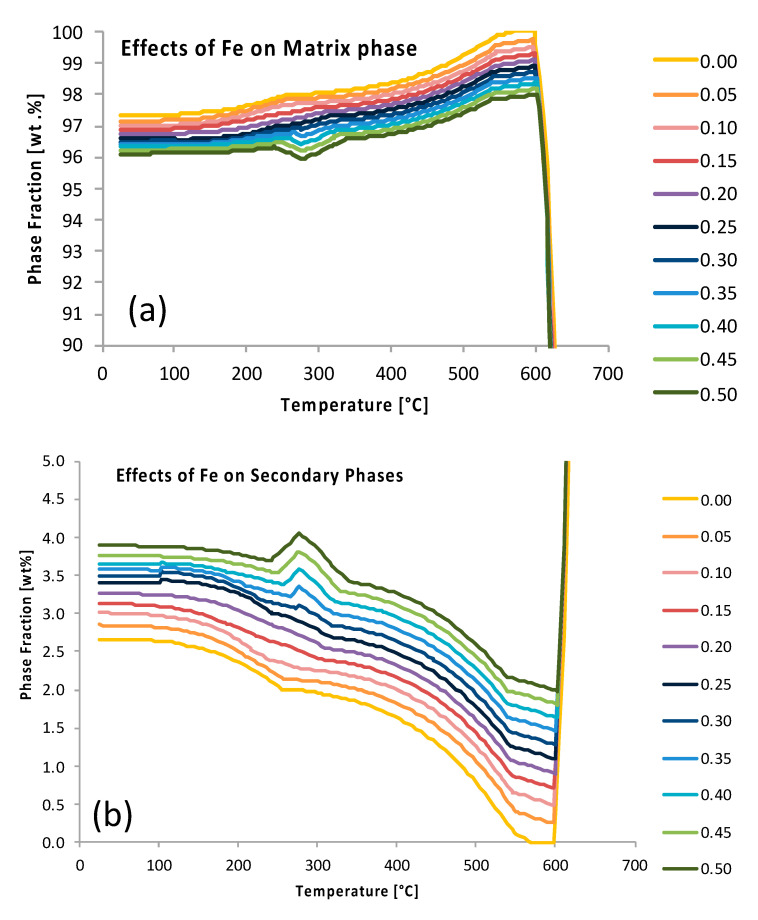
Data associated with the ψs for iron on the matrix phase present in Al 6061 at 25 °C are presented in (**a**). Data associated with the ψs for iron for the summation of the secondary phases present in Al 6061 at 25 °C are presented in (**b**).

Analysis of Figure 11 allows for the determination of which element, manganese or magnesium, has a greater effect on the amount of the strengthening theta phase found, which is associated with 2xxx series aluminum alloys. From Figure 11, for heat treatments at 300 °C for 1 h and 10 h, the ψ of manganese was −49.7 and −40.9, respectively, and 1.8 and 1.6 for magnesium, respectively. This indicates that the addition of manganese to an Al-Cu system actually reduces the amount of theta, while the addition of magnesium increases this amount. Further, the diameter of theta is reduced by the addition of manganese, as was shown in Figure 11c, while the diameter of theta is nearly constant with the addition of magnesium, as has been shown in Figure 11d. Further elaboration and analysis can be performed on the variation of the ψs as a function of composition and heat treatment processing conditions to determine the effects of additional alloying elements on the amount and size of other precipitate/dispersoid phases too. Thus, the presently alternative, non-6061-based EIF inspection speaks to the versatility of the ψ beyond the single case discussed, in terms of Al 6061, throughout the majority of the present paper. The insights gleaned from the data presented in Figure 11 and analyzed accordingly also substantiates the utility of ψ-inspection for kinetic phenomena as well. Figure 12 presents the data associated with the ψs for iron on the Al matrix phase present in Al 6061 at 25 °C in Figure 12a and the data associated with the ψs for iron for the summation of the secondary phases present within Al 6061 at 25 °C, as presented in Figure 12b.

**Figure 13:**
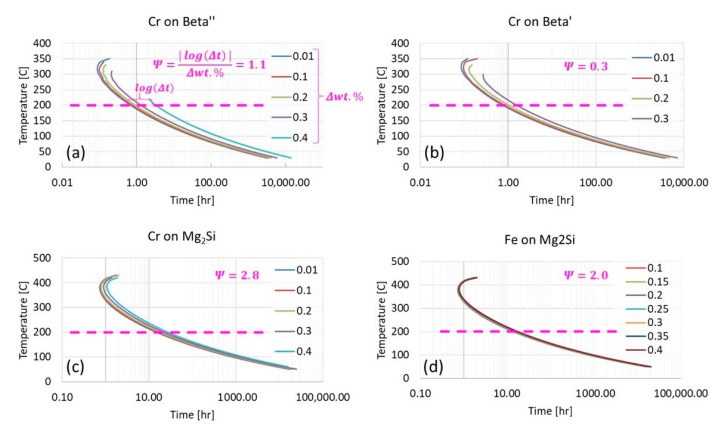
The influence of chromium upon β″ and β′ are presented in (**a**,**b**), respectively. As for chromium and iron in relation to Mg_2_Si, chromium was found to have a greater ψ at 2.8, as noted in (**c**), versus that of 2.0 for the effect of iron on Mg_2_Si, as shown in (**d**), under the same conditions.

**Figure 14:**
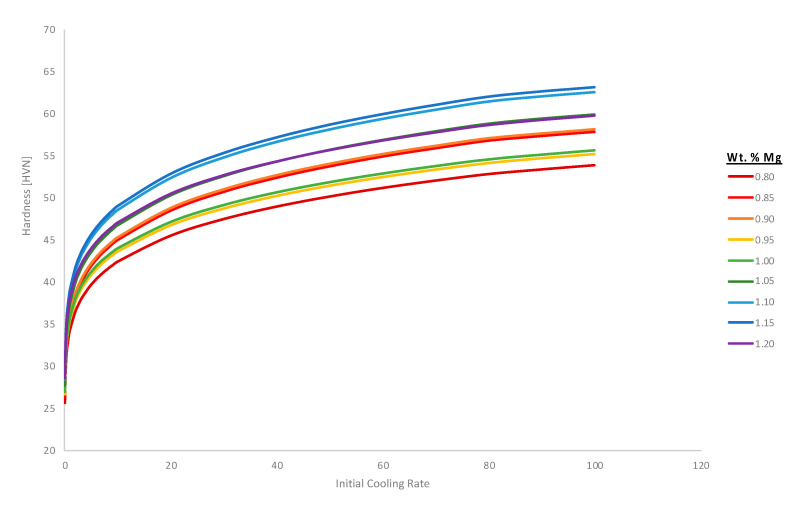
The hardness associated with the variation of magnesium content in the Al 6061 alloy, as a function of the initial solidification cooling rate, as presented herein, upon solidifying from a temperature of 700 °C.

Having returned to Al 6061 in Figure 12, after tangentially considering the ternary Al-Cu-(Mn,Mg) systems in Figure 11, Figure 13 continues to pursue enhanced ψ analysis of Al 6061 via kinetic modeling modalities. As a result, ψ-like assessments were successfully applied to TTT diagrams for β″, β′, and Mg_2_Si at 200 °C from the vantage point of increments of 0.01 wt.% chromium to 0.3 wt.% or 0.4 wt.% Cr depending upon on the metastable or stable phase(s) under consideration. At the same time, the ψ associated with the effect of iron content on the Mg_2_Si TTT behavior was provided for comparison with chromium’s respective influence. From ψ-inspection of Figure 13a, in comparison with Figure 13b, which corresponds with the influence of chromium upon β″ and chromium upon β′, respectively, chromium was found to have a more significant influence upon β″ formation than that of β′ formation with a ψ nearly three times greater than the ψ associated with chromium’s effect on β′. As for chromium and iron in relation to Mg_2_Si, chromium was found to have a greater ψ at 2.8 versus that of 2.0 for the effect of iron on Mg_2_Si under the same conditions. Remarkably, the observation that chromium attains a greater ψ in relation to Mg_2_Si as compared with the influence of iron on Mg_2_Si is consistent with relatively recent experimental findings too. Stated otherwise, the fact that $\psi_{Fe,\;200\;{}^{\circ}C}^{Mg_{2}Si}<\psi_{Cr,\;200\;{}^{\circ}C}^{Mg_{2}Si}$ is consistent with Kassner et al.’s assertion that “chromium may increase Mg_2_Si concentration and nucleation rate in 6xxx series aluminum alloys during a study of the quench sensitivities of 6061-T6 and other 6xxx series alloys” in [31]. Finally, Figure 14 computes the hardness associated with the variation of magnesium content in the Al 6061 alloy and as a function of the initial solidification cooling rate too.

**Figure 15:**
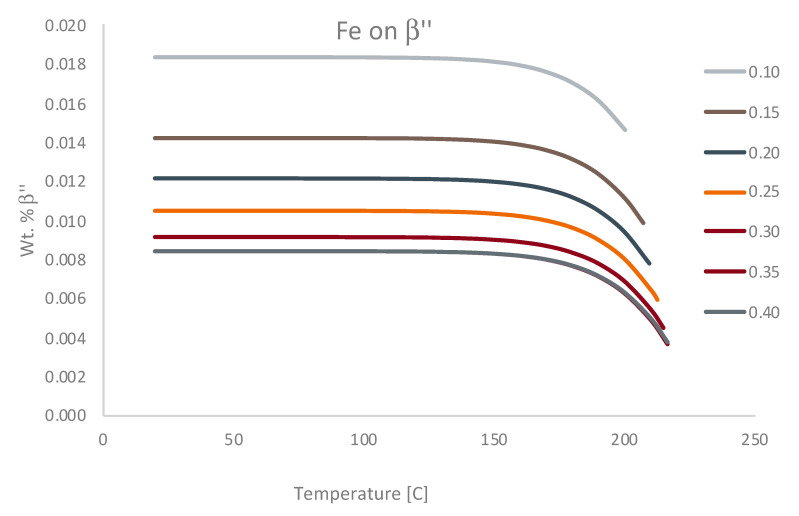
Phase fractions of β″ phase as a function of varying iron content over a range of temperatures.

In addition to calculating the EIF for equilibrium phases, EIFs can also be calculated for metastable phases. Currently, due to the limitations of the kinetic and thermodynamic software utilized, the only way to calculate phase fraction diagrams for metastable phases requires the suspension of phases suspected or known to be absent at desired conditions. An example of the phase fraction plot for a metastable phase is shown in Figure 15. The metastable phase is the β″ phase, which is a metastable precursor to the stable Mg_2_Si phase. This approach is obviously intrinsically undesirable due to the need for previous knowledge about the system under consideration. This knowledge may be acquired by experimental observation or alternatively, kinetic diagrams can nevertheless be calculated to determine if certain phases have enough time to form during solidification (via CCT diagrams) or during aging or heating (via TTT diagrams).

## 5. Consideration of Negative Elemental Impact Factors

As discussed within the aforementioned sections of the present research article, negative ψs are possible and alloying elements with a negative ψ value inform materials engineers or metallurgists with the knowledge that the addition of an element with such a ψ will decrease the amount of the equilibrium or metastable phase present with respect to the temperature and/or time associated with the modeled system. The same is true for kinetic ψs and property-based ψs wherein a negative ψ for a given element, in relation to a property, or microstructural constituent, and its’ characteristics, means that the element will deleteriously influence the material property under investigation, or reduce the diameter of a precipitate or intermetallic compound within the material under relevant temperatures and times, for example. Though discussed only briefly in the section of the present manuscript titled “Property, Kinetic, and Metastable Elemental Impact Factors”, the reader will have noticed that negative ψs were determined with respect to the system studied and presented as part of Figure 11. Recall that the ψ as a function of manganese content and time at 300 °C was −49.7 and −40.9 for 1 h and 10 h of thermal processing times for an Al-Cu-Mn ternary system with balanced aluminum content, 4.0 wt.% Cu, and 0.001–1.99 wt.% Mn. This was computed in relation to the vol.% of theta formed in such systems under said processing conditions.

**Figure 16:**
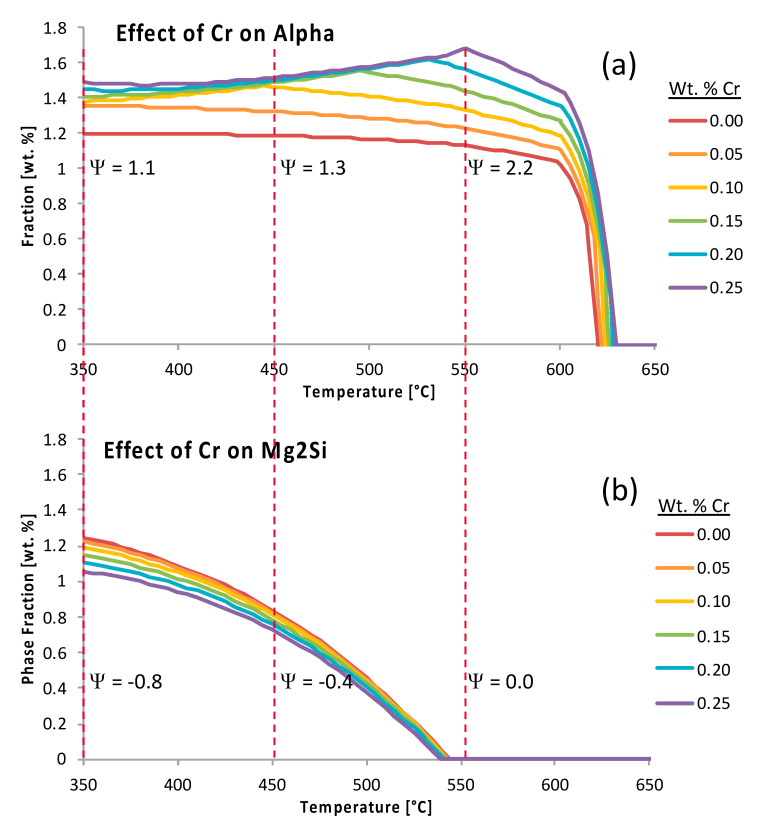
(**a**) The fractional weight percent of alpha as a function of temperature and the weight percent of chromium relative to the Al 6061 system. (**b**) The effect of chromium on the phase fraction of Mg_2_Si as a function of said alloying element and temperature**.**

With the aforementioned in mind, additional consideration of negative ψs will be considered hereafter. Accordingly, Figure 16 presents the fractional weight percent of alpha, as a function of temperature, the weight percent of chromium relative to the Al 6061 system, the effect of chromium on the phase fraction of Mg_2_Si as a function of said alloying element, and the temperature as well. While $\psi_{Cr,\;350\;{}^{\circ}C\;}^{\alpha }$ was 1.1, $\psi_{Cr,\;450\;{}^{\circ}C\;}^{\alpha }$ was 1.3 and $\psi_{Cr,\;550\;{}^{\circ}C\;}^{\alpha }$ was 2.2 (all positive ψs) the Mg_2_Si phase fraction versus temperature curve, as a function of chromium content, exemplifies the case scenario wherein negative ψs were observed until reaching a value of zero between approximately 550 and 650 °C. Specifically, $\psi_{Cr,\;350\;{}^{\circ}C\;}^{Mg_{2}Si}$ was −0.8, $\psi_{Cr,\;450\;{}^{\circ}C\;}^{Mg_{2}Si}$ was −0.4 and $\psi_{Cr,\;550\;{}^{\circ}C\;}^{Mg_{2}Si}$ was 0.0, in accordance with the embedded values shown in Figure 16.

**Figure 17:**
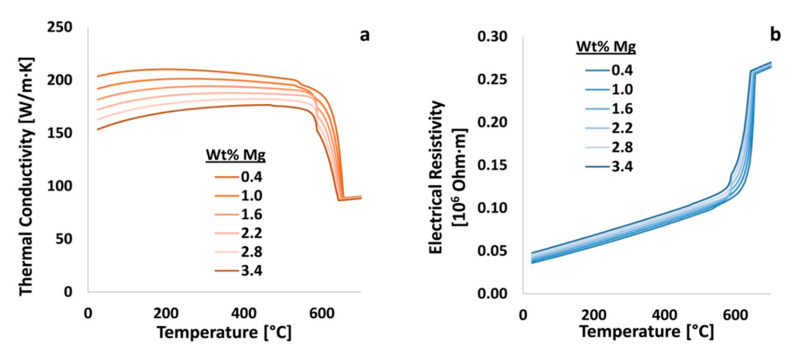
(**a**) The effect of the magnesium content on the thermal conductivity of Al 6061. (**b**) The effect of the magnesium content on the electrical resistivity of Al 6061.

As illustrated through the consideration of Figure 16 and prior figures detailed earlier, EIF analysis also includes the distinction between positive and negative phase–element and element–property effects. For instance, the addition of one element may cause an increase in the quantity of one phase while reducing the amount of a different phase. On the other hand, one element may cause an increase in the magnitude of a property while decreasing another. The way in which one element may have a deleterious effect upon the performance of a particular system, which was readily identifiable via the consultation of the appropriate ψ of interest, was exemplified herein. In Figure 17, magnesium was varied in Al 6061 from 0.4 to 3.2 wt.% and the resulting thermal conductivity and electrical resistivity of the material was calculated using JMatPro. Shortly thereafter, the resulting thermal conductivities and electrical resistivities, as a function of temperature, were assessed thereafter in terms of the elements effects on said properties as quantified by way of ψ.

An example of property versus temperature data, as a function of a particular alloying and elemental content that results in negative ψs, may be observed by way of consulting Figure 17a. The calculated and negative ψs for magnesium with respect to the thermal conductivity of Al 6061 at 20 and 450 °C were −8.2 and −4.5, respectively. However, the calculated and positive ψs for magnesium on the electrical resistivity, at the same temperatures, are 3.8 and 1.8, respectively. Therefore, from the quantification achieved via EIF analysis, it stands to reason that as the content of magnesium increases, the thermal conductivity of Al 6061 decreases. Inversely, an increase in the content of magnesium increases the electrical resistivity of the 6xxx series alloy considered herein and throughout the present work. Further, as the temperature increases, magnesium has a lower effect on both the thermal conductivity and electrical resistivity of the system.

**Figure 18:**
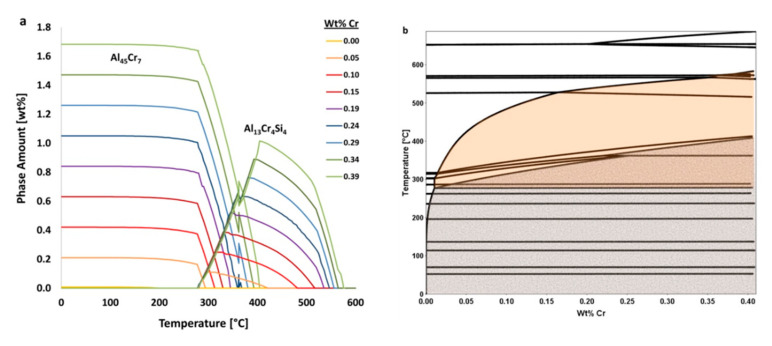
(**a**) The resulting phase fractions of Al_13_Cr_4_Si_4_ and Al_45_Cr_7_ as a function of chromium content. (**b**) The isopleth of Al 6061 with varying chromium contents. The orange shaded region in (**b**) represents the presence of Al_13_Cr_4_Si_4_, whereas the grey shaded region in (**b**) represents Al_45_Cr_7_.

Analogous analysis exemplifies an additional use of ψs for phases; that is, the reduction or even removal of deleterious phases in a material, which can occur by way of adding elements with a negative ψ in relation to said phase. Conversely, elements with a positive ψ, in such a scenario, could be used to increase the number of desired phases. To depict the physical representation of ψ, the utilization of the calculated isopleth for the average composition of Al 6061, as a function of chromium content, is shown in Figure 18. The orange and gray shaded regions in Figure 18b represent ranges where Al_13_Cr_4_Si_4_ and Al_45_Cr_7_ are respectively present at equilibrium. Between approximately 300 and 400 °C, Al_13_Cr_4_Si_4_ converts to Al_45_Cr_7_ upon cooling. Furthermore, the solubility of chromium in the FCC matrix increases with temperature and accounts for the balance of chromium in the system.

**Figure 19:**
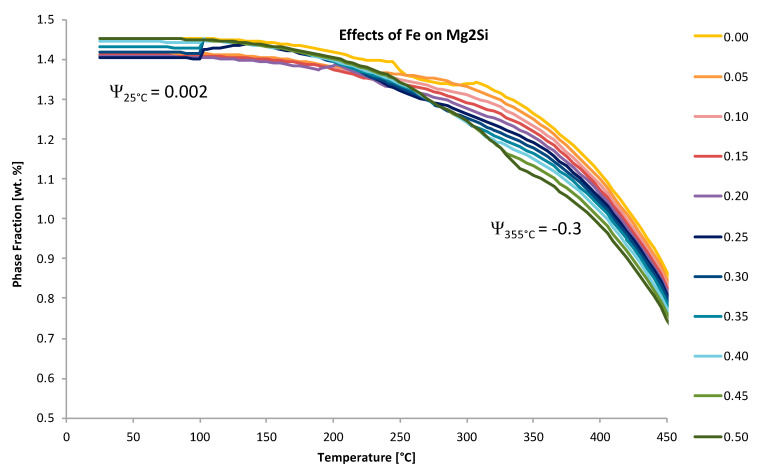
Imposes the relative impact of iron on Mg_2_Si as a function of varied iron content and temperature for a phase fraction versus temperature plot.

Another example of the differentiation between positive and negative EIF’s is given in Figure 19. Figure 19 depicts the way in which the equilibrium phase fraction, or the fractional, weight percent of Mg_2_Si, as a function of the temperature as well as the weight percent of iron relative to the alloyed aluminum system under consideration. Clearly, the respective EIF calculated at a temperature of 25 °C for Fe maintained a value that reveals a far less deleterious influence of Fe on Mg_2_Si than that of Fe at 355 °C.

## 6. Discussion of the Elemental Impact Factor and Physical Implications

**Figure 20:**
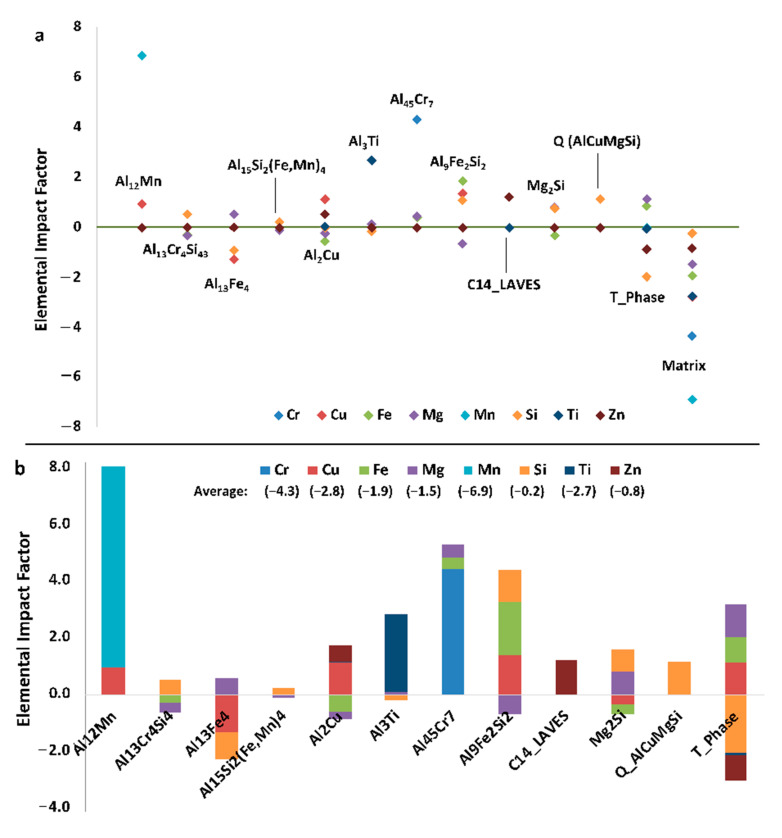
ψ values for Al 6061 at 25 °C. (**a**) The compilation of the ψs for the matrix and most abundant secondary phases present in Al 6061 at 25 °C. (**b**) The cumulative ψs for each equilibrium phase and element in Al 6061. For (**a**,**b**), chromium is identified with the blue color, copper is associated with the red color, iron is identified with the green color, magnesium is associated with the purple color, manganese is identified with the light blue color, silicon is associated with the orange color, titanium is identified with the dark blue color, and zinc is associated with the brown color in the plots.

Relative EIFs can also be a valuable method to study the effects of composition and/or temperature variation. Figure 20a contains the EIFs for the matrix and secondary phases for each element in Al 6061 at 25 °C. Here it is clearly depicted which elements influence, positively or negatively, the amount of equilibrium phases present. Some of these results are intuitive: the addition of magnesium and silicon both have positive influences on the formation of Mg_2_Si. This analysis also allows for the detection of elemental influences that are arguably much less obvious. For example, copper and iron have a negative effect on the amount of the Mg_2_Si phase and magnesium has a negative effect on the Al_9_Fe_2_Si_2_ phase despite none of those elements being present in the standard stoichiometries associated with those respective phases. Similarly, manganese has a positive effect on Al_45_Cr_7_ and copper has a positive effect on Al_12_Mn. It is evident that the addition of any of the elements, with the exception of aluminum, decreases the amount of the matrix phase, and therefore increases the number of secondary phases. The Mg_2_Si phase is also more easily influenced by compositional changes than the Al_3_Fe phase and therefore may require more significant compositional changes to be mitigated or created in accordance with the resultant findings from EIF-based analysis.

**Figure 21:**
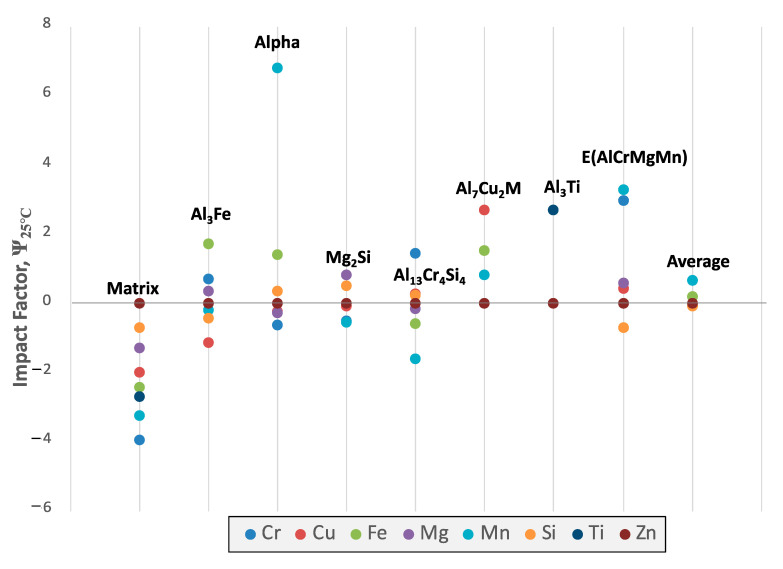
Compilation of the ψs for the matrix and most abundant secondary phases present in Al 6061 at 25 °C.

Considering the fact that the mentioned motivation and purpose surrounding the presentation of Figure 20 was to concurrently present the directly computed ψs for the matrix and secondary phases present in the Al 6061 alloy system, equilibrated at 25 °C and presented in Figure 20a as compared with the cumulative ψs for each equilibrium phase and element in the Al 6061 system defined (see Figure 20b), Figure 21 presented the standard ψs for the phases associated with each element at the same temperature for additional clarity. Figure 21 clearly depicts which elements positively and negatively influence the amount of equilibrium phases residing within the system. One take away from a more focused inspection of Figure 21, for example, is that the addition of any element will not decrease the amount of the Al_7_Cu_2_M phase present at room temperature.

**Figure 22:**
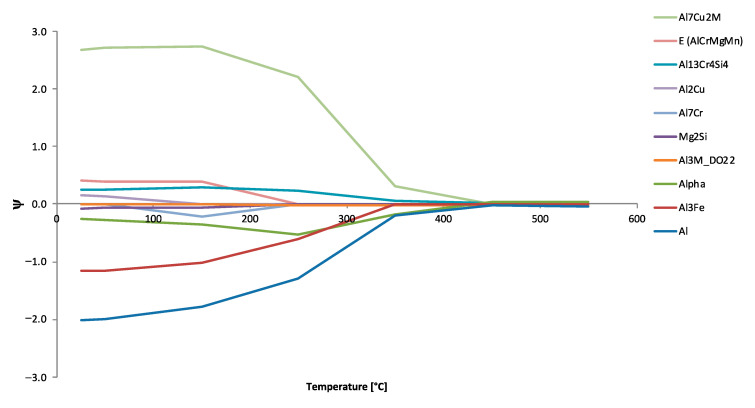
Plots the particular ψs for the specific alloying element copper for all of the phases identified in Figure 22’s legend as function of equilibrium temperature.

While Figure 20 and Figure 21 successfully highlighted the ψ relations and magnitudes associated with the alloying elements and phases studied for Al 6061 and assuming equilibrium conditions at 25 °C, Figure 22 plots the particular ψs for the specific alloying element copper with respect to all of the phases identified in Figure 22’s legend as function of equilibrium temperature. A global trend is clearly observed wherein the absolute value of a given EIF associated with copper decays towards zero influence as the equilibrium temperature of the system approaches the homogenization, solutionization, and melting temperature of Al 6061. One may additionally recognize the fact that $\psi_{Cu,T}^{\alpha }$ remains negative over the temperature range evaluated, ultimately reaching a ψ value of −0.25 at 25 °C with respect to copper. Copper is also found to have a negative ψ associated with Al_3_Fe, the aluminum matrix, Al_7_Cr at 150 °C and Mg_2_Si to start. As for the positive copper ψs, Al_2_Cu, Al_13_Cr_4_Si_4_, E and Al_7_Cu_2_M were phases that benefited from copper prior to the ψ versus temperature curves decay towards zero.

**Figure 23:**
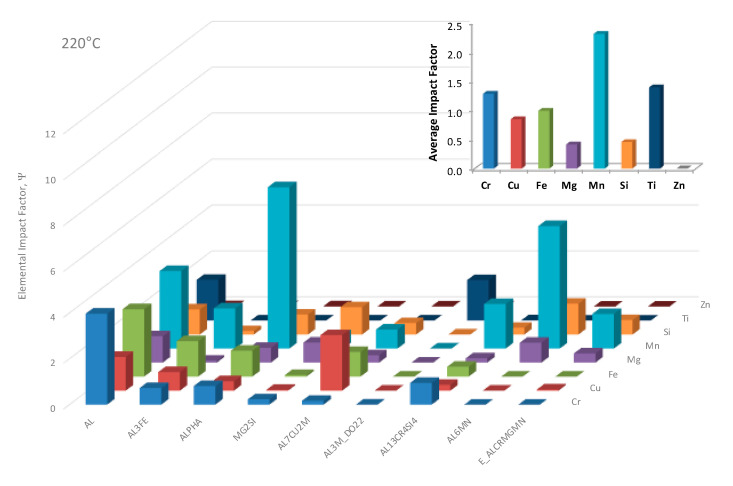
A multifaceted presentation of ψs associated with given combinations of alloying elements in the Al 6061 system of interest and the equilibrium phases predicted at 220 °C, which reflects the heat-treatable artificial aging temperature regime.

Having taken a more robust look into the ψs associated with copper as an alloying element, we may once again turn towards a multifaceted presentation of ψs associated with given combinations of alloying elements in the Al 6061 system of interest and the equilibrium phases predicted at 220 °C, which reflects the heat-treatable artificial aging temperature regime for the aluminum alloy considered in large part herein. Said assessment was presented in Figure 23. Additional analysis of the EIFs concerns the average ψs associated with each element and phase studied as part of Figure 23 at 220 °C too. This plot was embedded in the upper right corner of Figure 23. As a result, one may note that manganese, titanium and chromium maintained the greatest average ψs associated with equilibrium phases at 220 °C, whereas zinc, magnesium and silicon maintained the least impactful average ψs.

**Figure 24:**
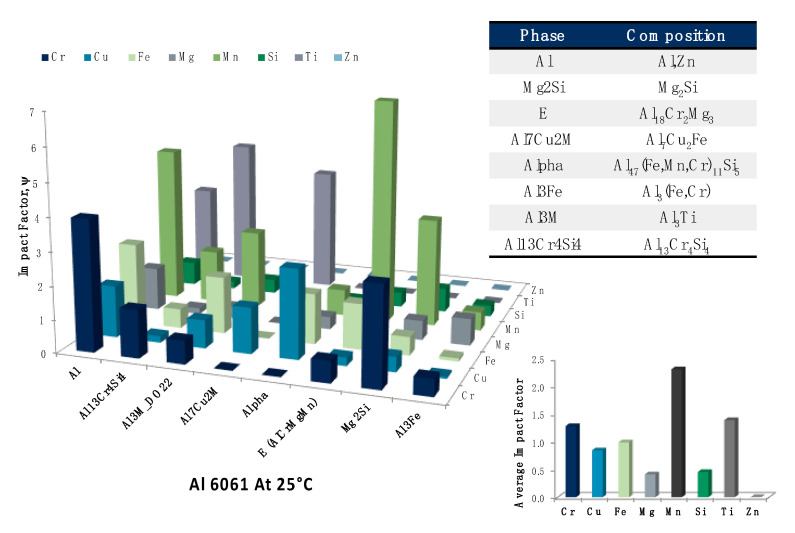
Presentation of the temperature associated with the known phenomena of natural aging in lightweight aluminum alloys. Once again, under equilibrium conditions, one may note that manganese, titanium and chromium maintained the greatest average ψs associated with equilibrium phases at 25 °C, whereas zinc, magnesium, and silicon, maintained the least impactful average ψs.

Consistent with the rationale associated with Figure 23, Figure 24 presents a similar analysis of ψs and average ψs for the same pairing of alloying elements and equilibrium phases found in Figure 23. However, unlike Figure 23, which dealt with a temperature in the artificial heat-treating or thermal processing temperature regime, Figure 24 entertains the temperature associated with the known phenomena of natural aging in lightweight aluminum alloys. Once again, and in terms of the EIF approach/analysis, under equilibrium conditions, one may note that manganese, titanium and chromium maintained the greatest average ψs associated with equilibrium phases at 25 °C, whereas zinc, magnesium and silicon maintained the least impactful average ψs. The table embedded in Figure 24 was provided to highlight the actual elements that could be associated with a given nominal phase, in a substitutional manner, even if a given phase’s defined stoichiometric ratios do not explicitly include other prospective elements present.

**Figure 25:**
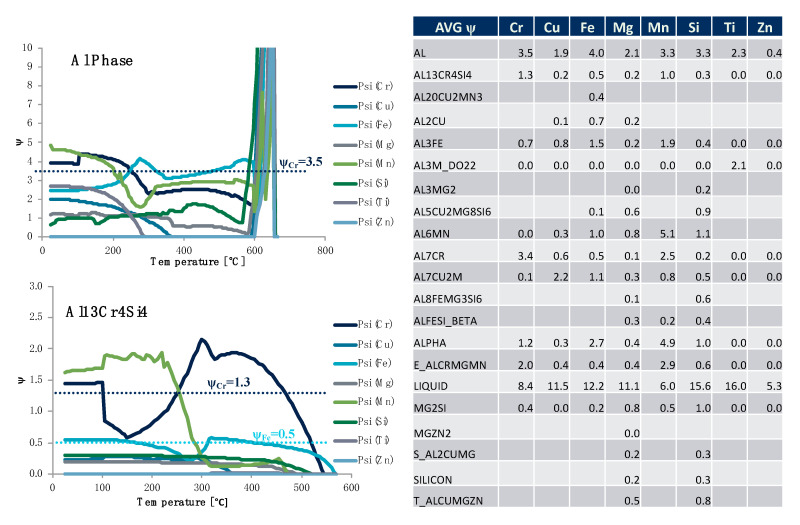
The ψs associated with each of the alloying elements for the matrix aluminum FCC matrix or base phase and the Al_13_Cr_4_Si_4_ phase as a function of temperature. At the same time, a table presenting the average ψs computed for the particular phase–element combinations studied over the range of temperatures considered in silico is presented in Figure 25 as well.

While Figure 23 and Figure 24 complimented one another, Figure 22 is consistent with the rationale associated with Figure 25. While Figure 22 looked at particular ψs for the specific alloying element of copper for all of the phases identified in Figure 22’s legend as function of equilibrium temperature, Figure 25 looks at the ψs associated with each of the alloying elements for the matrix aluminum base phase and Al_13_Cr_4_Si_4_ phase as a function of temperature. In fact, all of the ψs for the alloying elements computed are plotted against temperature for each of the phases in Figure 25. At the same time, a table presenting the average ψs computed for the particular phase–element combinations studied over the range of temperatures considered in silico is presented in Figure 25 as well. For example, we may observe that the average $\psi_{Cr}^{Al\left(FCC\right)}$ was 3.5, whereas the average $\psi_{Cr}^{Al_{13}Cr_{4}Si_{4}}$ was 1.3.

**Figure 26:**
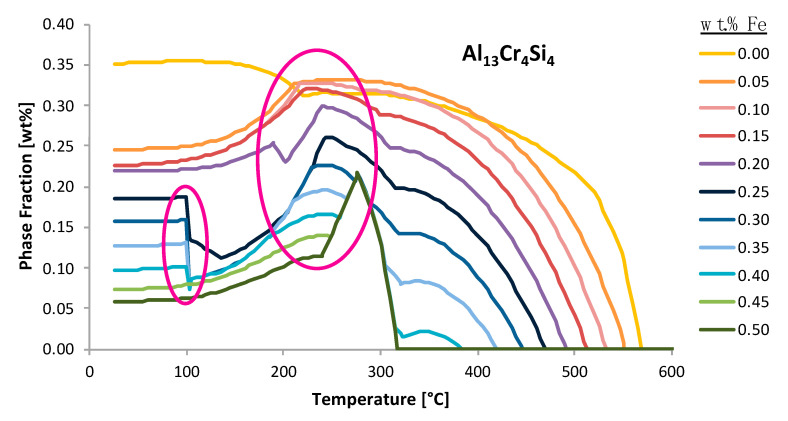
The phase fraction of Al_13_Cr_4_Si_4_ as a function of temperature and iron content for demonstrative purposes.

Throughout the figures presented so far, Figure 2, Figure 5, Figure 6, Figure 10, Figure 12, Figure 18 and Figure 19 capture instances wherein the phase fraction content as a function of a set of alloying elements will suddenly increase or decrease at a specific temperature in a discontinues and discretized manner. To clearly appreciate the abrupt changes being referred to herein, Figure 26 presents the phase fraction of Al_13_Cr_4_Si_4_ as a function of both the temperature and the iron content for demonstrative purposes. As can be seen in the first semi-transparent and pink oval shape imposed upon the Al_13_Cr_4_Si_4_ phase fraction versus temperature plot as a function of iron weight percent in the system, one may observe the aforementioned case scenario surrounding the sudden decrease in the weight percent phase fraction value at a specific temperature whilst also appearing in a discontinuous manner. On the other hand, the second semi-transparent, larger and pink oval shape imposed upon the same curve enables the reader to better appreciate an alternative increase, which is abrupt and sudden, but can be considered less qualitatively discretized than the first occurrence. To properly discern the physical implications surrounding EIFs, one must develop an appropriate explanation for such incidences.

**Figure 27:**
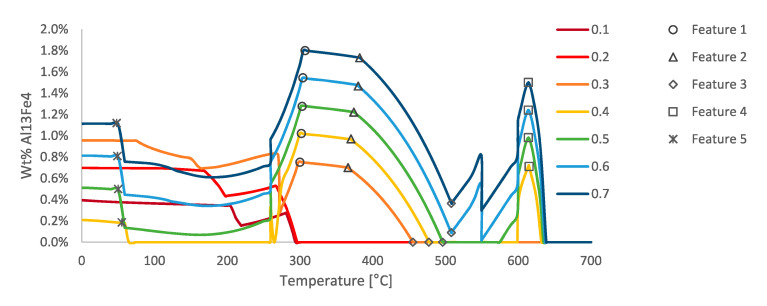
Effect of varying the content of iron on the equilibrium Al_13_Fe_4_ phase amount. The features are identified in the equilibrium isopleth in the following figure.

**Figure 28:**
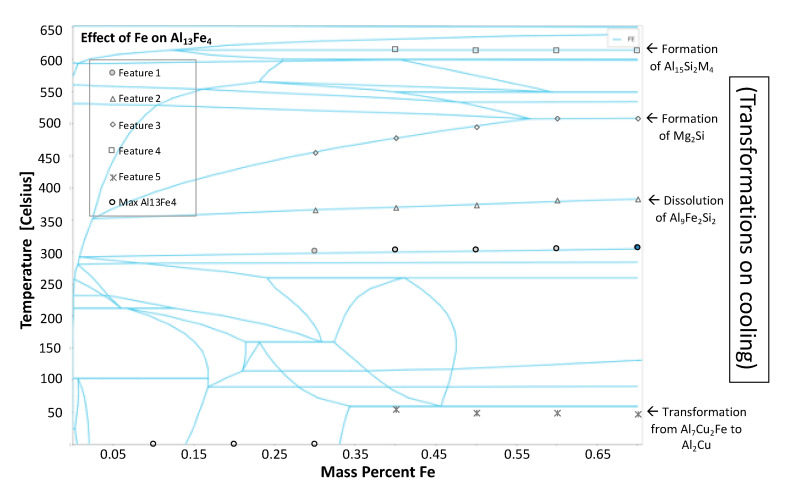
Equilibrium isopleth of Al 6061 with varying amounts of iron. Features 1–5 are corresponding to the behavior of equilibrium phases shown in the previous figure.

As was just alluded to it is important to understand the physical meaning behind the behavior of the phases shown in Figure 26, for example. One example of such behavior is observed in the phase fraction diagram in Figure 27 for the Al_13_Fe_4_ intermetallic phase fraction versus temperature curves as a function of varied iron contents. The abrupt changes in the phase fractions can be correlated to phase boundaries shown in Figure 28. The phase changes corresponding to these changes are given as follows: Feature 1 in Figure 27 and Figure 28 corresponds with the maximum amount of Mg_2_Si that can form at 300 °C; Feature 2 corresponds with the dissolution of Al_9_Fe_2_Si_2_ at 350 °C; Feature 3 corresponds with the formation of Mg_2_Si between 450 and 500 °C; Feature 4 corresponds with the formation of Al_15_Si_2_M_4_ at 600 °C; and Feature 5 corresponds with the transformation from Al_7_Cu_2_Fe to Al_2_Cu.

## 7. Prospective Applications and Limitations of the Elemental Impact Factor

The EIF is a quantitative method to determine the significance a given element has on any phase or property at a specific temperature, time and condition. Regarding phase formation, the EIF may be most useful when there are already known or identified beneficial or deleterious phases present in a given system. Some comments about practical applications of the EIF are listed below:

- When phases are identified as deleterious to a materials system by reducing its performance, elements with a negative EIF (meaning the addition of that element decreases the amount of phase under investigation) could be added to the system.
  ○ This can include a combination of elements or the addition of a foreign element not already in the system.
  ○ This is especially beneficial in cases where undesired phases are caused by the presence of impurity elements which cannot be removed from the alloy.
- Elements with a positive EIF were shown herein to increase the amount of phase under consideration. If possible, these elements could be reduced from the alloy during production to avoid the deleterious phase formation, for example.
- For the formation of desired phases, elements with a positive EIF can be increased to promote the amount of the desired phase. On the other hand, elements with a negative EIF can potentially be decreased, though the addition of elements is typically easier than that of reduction, at least in the case of commercially available materials.
- The addition of elements not traditionally present in the alloys being used may be examined with the EIF to determine potentially eligible elements, which may increase or decrease the amount of desired or deleterious constituents.

In the case of the examples presented, the desired and/or undesired phases would ideally already be known in order to achieve the greatest success with the EIF methodology. Such knowledge often requires an analysis of the literature of relevance and/or an experimental investigation into the material system and its failure mechanisms. Once these desired and/or undesired phases are known, the benefits of the EIF modality of analysis can be significant. As with many computational models and/or tools the time, resources, and monetary cost from modeling as opposed to experimentation can be drastically reduced. Although models cannot be a complete substitution for experiments, the EIF method can be used to determine calculated candidate chemical compositions for novel materials, among other uses.

Of course, this EIF approach has limitations and exceptions. First, phase stoichiometry is not accounted for independently, resulting in phase predictions occasionally showing discontinuous behavior at specific compositions. Further, at certain compositions, phase fractions actually cross one another, resulting in an inaccurate EIF at these abnormal points. Comparing the phase fraction plots for numerous alloys, data trends yield approximately 90% of content and temperature regions accurately represented by the EIF calculations.

## 8. Concluding Remarks and Future Work

The EIF can be used for numerous materials science endeavors, integrated and advanced metallic materials manufacturing, and materials engineering applications including:

- When deleterious phases are identified, the EIF can be used to identify alloy design processes to mitigate or even eliminate the undesirable phases through the addition or removal of elements.
- Beneficial phases may be formed by the addition, or even elimination, of other alloying elements in accordance with the EIF methods framework.
- General analysis of all the elements and phases over extended temperature ranges can be performed to generate a greater understanding of the materials system employed.
- The effect of alloy composition on material properties can be efficiently predicted.
- To improve desired properties, elements with a positive EIF should be added, while a reduction in properties will result from elements with a negative EIF.
- Metastable phases can be studied as a function of composition and heat treatment conditions, times and temperatures. Effects on precipitate amount, size, nucleation rate, critical radius, and nuclei density can be effectively predicted via EIF inspection too.

In all cases, the efficiency of chemical alteration in alloy design is significantly improved. Use of the EIF method presented herein results in a reduction in time, cost, and resources compared with addressing these areas with conventional experimental and modeling techniques.
